# Genome and Functional Characterization of Colonization Factor Antigen I- and CS6-Encoding Heat-Stable Enterotoxin-Only Enterotoxigenic *Escherichia coli* Reveals Lineage and Geographic Variation

**DOI:** 10.1128/mSystems.00329-18

**Published:** 2019-01-15

**Authors:** Tracy H. Hazen, Sushma Nagaraj, Sunil Sen, Jasnehta Permala-Booth, Felipe Del Canto, Roberto Vidal, Eileen M. Barry, Jacob P. Bitoun, Wilbur H. Chen, Sharon M. Tennant, David A. Rasko

**Affiliations:** a Institute for Genome Sciences, University of Maryland School of Medicine, Baltimore, Maryland, USA; b Department of Microbiology and Immunology, University of Maryland School of Medicine, Baltimore, Maryland, USA; c Programa de Microbiología y Micología, Instituto de Ciencias Biomédicas, Facultad de Medicina, Universidad de Chile, Santiago, Chile; d Instituto Milenio de Inmunología e Inmunoterapia, Facultad de Medicina, Universidad de Chile, Santiago, Chile; e Center for Vaccine Development, Institute for Global Health and Department of Medicine, University of Maryland School of Medicine, Baltimore, Maryland, USA; f Department of Microbiology & Immunology, Tulane University School of Medicine, New Orleans, Louisiana, USA; University of British Columbia

**Keywords:** *Escherichia coli*, comparative genomics, heat-stable toxin

## Abstract

Enterotoxigenic Escherichia coli (ETEC) is a significant cause of childhood diarrhea and is a leading cause of traveler’s diarrhea. ETEC strains encoding the heat-stable enterotoxin (ST) are more often associated with childhood diarrhea than ETEC strains that encode only the heat-labile enterotoxin (LT). Colonization factors (CFs) also have a demonstrated role in ETEC virulence, and two of the most prevalent CFs among ETEC that have caused diarrhea are colonization factor antigen I (CFA/I) and CS6. In the current report, we describe the genomes of 269 CS6- or CFA/I-encoding ST-only ETEC isolates that were associated with human diarrhea. While the CS6 and CFA/I ETEC were identified in at least 13 different ETEC genomic lineages, a majority (85%; 229/269) were identified in only six lineages. Complete genome sequencing of selected isolates demonstrated that a conserved plasmid contributed to the dissemination of CFA/I whereas at least five distinct plasmids were involved in the dissemination of ST and/or CS6. Additionally, there were differences in gene content between CFA/I and CS6 ETEC at the phylogroup and lineage levels and in association with their geographic location of isolation as well as lineage-related differences in ST production. Thus, we demonstrate that genomically diverse E. coli strains have acquired ST, as well as CFA/I or CS6, via one or more plasmids and that, in some cases, isolates of a particular lineage or geographic location have undergone additional modifications to their genome content. These findings will aid investigations of virulence and the development of improved diagnostics and vaccines against this important human diarrheal pathogen.

**IMPORTANCE** Comparative genomics and functional characterization were used to analyze a global collection of CFA/I and CS6 ST-only ETEC isolates associated with human diarrhea, demonstrating differences in the genomic content of CFA/I and CS6 isolates related to CF type, lineage, and geographic location of isolation and also lineage-related differences in ST production. Complete genome sequencing of selected CFA/I and CS6 isolates enabled descriptions of a highly conserved ST-positive (ST^+^) CFA/I plasmid and of at least five diverse ST and/or CS6 plasmids among the CS6 ETEC isolates. There is currently no approved vaccine for ST-only ETEC, or for any ETEC for that matter, and as such, the current report provides functional verification of ST and CF production and antimicrobial susceptibility testing and an in-depth genomic characterization of a collection of isolates that could serve as representatives of CFA/I- or CS6-encoding ST-only ETEC strains for future studies of ETEC pathogenesis, vaccine studies, and/or clinical trials.

## Abstract (precis)

Comparative genomics and functional characterization were used to analyze a global collection of CFA/I and CS6 ST-only ETEC isolates associated with human diarrhea, demonstrating differences in the genomic content of CFA/I and CS6 isolates related to CF type, lineage, and geographic location of isolation and also lineage-related differences in ST production. Complete genome sequencing of selected CFA/I and CS6 isolates enabled descriptions of a highly conserved ST-positive (ST^+^) CFA/I plasmid and of at least five diverse ST and/or CS6 plasmids among the CS6 ETEC isolates. There is currently no approved vaccine for ST-only ETEC, or for any ETEC for that matter, and as such, the current report provides functional verification of ST and CF production and antimicrobial susceptibility testing and an in-depth genomic characterization of a collection of isolates that could serve as representatives of CFA/I- or CS6-encoding ST-only ETEC strains for future studies of ETEC pathogenesis, vaccine studies, and/or clinical trials.

## INTRODUCTION

Enterotoxigenic Escherichia coli (ETEC) is a leading cause of severe diarrheal illness each year among children under 5 years of age (1) and is also a leading cause of traveler’s diarrhea among adults (2, 3). ETEC isolates are characterized by the heat-labile enterotoxin (LT) and/or the heat-stable enterotoxin (ST) (3–5). The human ST (STh) variant is the most prevalent ST toxin associated with human diarrhea, while the porcine ST (STp) variant was originally identified in ETEC associated with porcine diarrhea and is more prevalent among ETEC isolates from animals (2, 6). ETEC colonization factors (CFs) also play an important role in the ability of ETEC to cause disease by facilitating adherence to the intestinal epithelium (3, 7). At least 27 CFs have been functionally described to date (7, 8); however, the most prevalent CFs are colonization factor antigen I (CFA/I) and CS1 to CS6 (2, 3, 7, 9–11).

The Global Enteric Multicenter Study (GEMS), a large-scale (LS) prospective case-control study investigating the causes of childhood diarrhea in countries of Africa and Asia (12), identified ETEC as one of the top four leading causes of moderate-to-severe diarrhea (MSD) in children under 5 years of age (1). A critical finding of the GEMS investigation was that ST-encoding ETEC isolates (with or without the copresence of LT) were significantly associated with MSD whereas ETEC isolates that encoded only LT were not associated with MSD (1, 67). These findings corroborate the idea of the epidemiological significance of diarrhea associated with ST-encoding ETEC isolates, which have been considered a public health concern since their initial description in the 1970s (13).

Although ST-only ETEC strains are a significant global childhood health concern, there is currently no approved vaccine for this important diarrheal pathogen, and previous controlled human infection model (CHIM) studies performed with ETEC utilized only a limited number of isolates (14–19), most of which were selected based on phenotypic data without the interrogation of genomic information. Thus, in the current study we used comparative genomics and functional characterization to examine the diversity of ST-only ETEC isolates, focusing on isolates with CFA/I or CS6, as these are two of the most prevalent CF types historically associated with human diarrheal illness and were found to be similarly prevalent among cases in GEMS (2, 3, 7, 9–11, 67). We characterized the genomes of 269 ST-only ETEC isolates from two well-described and geographically diverse ETEC collections, including 162 CFA/I-encoding ST-only ETEC isolates and 107 CS6-encoding ST-only ETEC isolates, here referred to as CFA/I ETEC and CS6 ETEC, respectively. Also, we used long-read sequencing to complete the genome assemblies of 20 CS6 ETEC isolates and 6 CFA/I ETEC isolates, to provide additional insight into the unique genomic content, including ST- and/or CF-encoding virulence plasmids, of representative CS6 ETEC and CFA/I ETEC isolates associated with human diarrheal illness.

## RESULTS

### Isolate selection and genome characteristics of the CS6 and CFA/I ETEC isolates.

To gain insight into the genomic diversity of the most prevalent groups of ST-only ETEC, we used a PCR-based approach to screen all of the ETEC isolates from the GEMS collection (1, 12), which were isolated from four countries in Africa and three countries in south Asia. To increase the geographic diversity of isolates to include three continents (Africa, Asia, and South America), we also included diarrheagenic ETEC isolates from Chile. In total, 1,194 ETEC isolates were examined, including 1,067 ETEC isolates associated with MSD from the GEMS collection and 127 diarrheagenic ETEC isolates from Chile. PCR-based detection of genes encoding ST and LT identified 355 ST-only ETEC isolates (293 from the GEMS collection and 62 from Chile) (67). An additional selection criterion applied prior to genome sequencing was to identify the CFA/I- or CS6-encoding ETEC isolates, which represent two of the most dominant CF types identified among the ETEC isolates in GEMS and other studies (2, 3, 7, 9–11).

Laboratory-based prescreening of the ETEC isolates led us to select and examine the genome contents of 269 unique ETEC isolates that encode either CFA/I or CS6 (162 CFA/I and 107 CS6 isolates) (see Table S1 in the supplemental material). The 269 CFA/I and CS6 genomes had sizes of 4.7 to 5.7 Mb and GC content of 50.09% to 50.97% (Table S1), which is consistent with previously sequenced ETEC genomes (20, 21). The CFA/I and CS6 genomes had 30 different predicted multilocus sequence types (MLST). However, 60% (162/269) of the ETEC genomes were one of two MLST sequence types (ST2332 and ST443), while 17 sequence types were represented by only a single genome (Table S1). The CFA/I and CS6 genomes were represented by 43 different serotypes (Table S1). As with the MLST results, eight serotypes were dominant (O128ac:H45, O115:H5, O114:H45, O128ac:H12, O71:H45, O148:H28, ONT:H45, and O114:H5) and represented 74% (199/269) of the genomes, while 26 of the serotypes were represented by a single genome (Table S1). Previous ETEC genome assemblies have contained as many as six plasmids in a single isolate (20, 21); therefore, it was not surprising that the number of predicted replicon types identified in each of the genomes ranged from 0 to as many as 8 (Table S1). The most prevalent plasmid replicons were IncFIB(AP001918) in 66% (177/269), IncFII(AY458016) in 32% (85/269), and IncFII(pCoo) in 21% (56/269) of the genomes (Table S1). The prevalence of IncFIB and IncFII plasmids is consistent with previous studies that have reported the association of E. coli virulence genes with these plasmid types (20, 22).

10.1128/mSystems.00329-18.5 TABLE S1 Genomes analyzed in this study. Download Table S1, XLSX file, 0.08 MB. Copyright © 2019 Hazen et al. 2019 Hazen et al. This content is distributed under the terms of the Creative Commons Attribution 4.0 International license.

### CS6 and CFA/I ETEC occupy diverse phylogenomic lineages.

**FIG 1:**
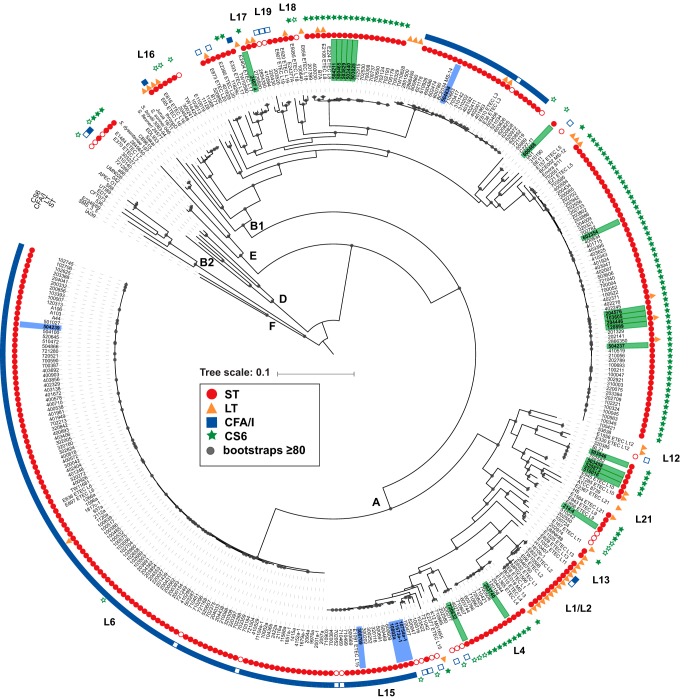
Phylogenomic analysis of the CFA/I ETEC and CS6 ETEC. The phylogeny was constructed from 231,031 conserved SNP sites per genome that were identified by comparison to reference genome E. coli isolate IAI39 (GenBank accession no. NC_011750.1). The tree scale indicates the distance of 0.1 nucleotide changes per site. Bootstrap values of ≥80 are indicated by gray circles. The presence of genes encoding ST, LT, CS6, and CFA/I is indicated by symbols adjacent to each genome name (see inset figure legend). The filled symbols indicate the genes that were identified by an initial PCR screen and also in the genome assembly, while an open symbol indicates genes that were detected by PCR but absent from the genome assembly. The CS6 ETEC isolates that were subjected to additional sequencing to generate complete genomes are indicated with a green rectangle around the isolate label, while the CFA/I ETEC isolates are indicated with a blue rectangle around the isolate label. The E. coli phylogroups are designated by letters (A, B1, B2, D, E, and F), while the previously described ETEC phylogenomic lineages are indicated by the designations L1 to L21 (with the exception of L14, for which we could not obtain a high-quality assembly for the references) (10).

Phylogenomic analysis demonstrated that the CFA/I and CS6 ETEC isolates are genomically diverse, with representatives in three (A, B1, and D) of the six E. coli phylogroups (Fig. 1). The 269 CFA/I ETEC and CS6 ETEC genomes were most prevalent in phylogroup A, with 58% (157/269) of the genomes, and phylogroup B1, with 40% (108/269) of the genomes, while only 1% (4/269) of the genomes were identified in phylogroup D (Fig. 1). Of the 269 ETEC genomes analyzed, 91% (245/269) were identified in 13 of the 21 previously described ETEC lineages (10) (Fig. 1). Although the CS6 genomes were present in eight lineages, 74% (80/107) of these genomes were present in only three lineages (L4, L5, and L8), and 51% of these genomes grouped in a single lineage (L5) (Fig. 1). The CS6 L5 isolates were from all geographic sites, demonstrating that this lineage is not geographically restricted (Table S1). Although the CFA/I genomes were identified in more than seven different lineages, 92% (149/162) were in only three lineages (L3, L6, and L15) (Fig. 1). Similarly to the CS6 ETEC genomes, more than half of the CFA/I genomes (67%; 109/162) were grouped in a single lineage (L6), and this lineage contained isolates from Chile and from all but one of the GEMS sample sites (Bangladesh) (Table S1).

### ST production levels differ by lineage but not by CF type.

**FIG 2:**
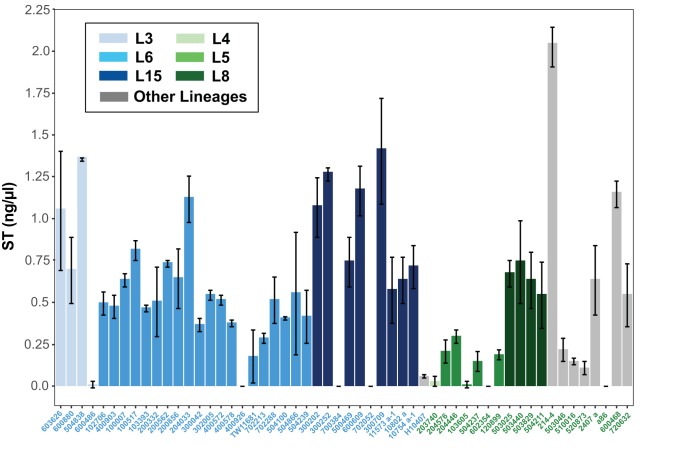
ST production by selected CFA/I- or CS6-producing ST-only ETEC isolates. ST production was measured by ST-mediated accumulation of cGMP (in picomoles per milliliter) in T84 cell monolayers cultured on 24-well tissue culture-treated plates. T84 monolayers were pretreated with the phosphodiesterase inhibitors zardaverine and vardenafil followed by the addition of 10 μl of ETEC cell culture supernatant from shaking cultures grown in 4AA medium or known masses of ST (BEI Resources; NR-50765). The values represent means and standard deviations of results from triplicate assays. The amount of cGMP in the supernatant of each of the ETEC isolates was calibrated to the amount of cGMP produced by treatment of T84 monolayers with 10 ng of purified STh to determine the amount of ST (in nanograms per microliter) produced by the ETEC isolates. The CFA/I ETEC isolates are indicated with blue isolate labels, while the CS6 ETEC isolates are indicated with green isolate labels (see inset legend). The bars are colored according to the phylogenomic lineage.

The presence of genes encoding ST among the ETEC isolates was confirmed via PCR and *in silico* analysis of their genome assemblies; however, we wanted to examine whether there is variability in the functional production of the ST toxin by selected CFA/I and CS6 isolates. We examined 35 CFA/I and 19 CS6 isolates for their ability to produce and secrete ST into culture medium using chemically defined 4AA media (23). ST binds to the intestinal guanylate cyclase C receptor, which is expressed on human colonic cell line T84 and stimulates the buildup of intracellular cyclic GMP (cGMP) as previously described (24). A range of ST-induced cGMP accumulation was observed from the CFA/I and CS6 isolate supernatants, suggesting that some isolates do not made significant ST while others made robust amounts of ST under the conditions examined (Fig. 2). Two of the ETEC isolates (a86 and 702052) had no detectable ST production and did not contain an STh or STp gene in their genome assemblies, suggesting that the ST-encoding plasmids were lost from these isolates. There were no significant differences with respect to the amount of ST produced by CFA/I isolates compared with CS6 isolates (Fig. 2). Also, there were no observed lineage-specific differences in ST production among the CFA/I isolates; however, the CS6 isolates exhibited lineage-specific differences in ST production (Fig. 2). The CS6 ETEC of lineage L8 produced more ST than the CS6 ETEC of lineage L5 (*P* < 0.001) (Fig. 2).

### CFA/I and CS6 ETEC genomes contain CF-, phylogroup-, and lineage-specific genes.

To determine whether there were any genes associated with particular lineages of CS6 ETEC or CFA/I ETEC, we used a gene-based approach to identify their shared and unique gene content. After excluding genomes that had LT genes or were missing the genes encoding ST, CFA/I, and CS6, we found that there were a total of 142 CFA/I genomes and 87 CS6 genomes for further analysis. We compared these genomes to each other as well as to a diverse collection of 37 ETEC reference genomes representing other CF types, which carry the genes for LT and/or ST (Table 1; see also Table S1). There were no genes in addition to the CS6-encoding genes that were present in all of the CS6 ETEC isolates and absent from the CFA/I ETEC isolates and only one gene in addition to the CFA/I genes that was present in all of the CFA/I genomes and absent from the CS6 genomes (Table 1; see also Table S2A and C). The additional gene that was unique to the CFA/I ETEC is identical to a region of previously sequenced ETEC isolate H10407 plasmid p948 that encodes CFA/I (GenBank accession no. FN649418.1).

**TABLE 1:** Summary of the gene-based comparisons of the CFA/I and CS6 genomes

Group 1^*a*^	Group 2	No. of genomes in group 1	No. of genomes in group 2	No. of gene clusters^*b*^		
				All genomes	≥50% genomes	≥1 genome
CFA/I	CS6	142	87	2	193	1,771
CS6	CFA/I	87	142	4	107	2,327
CFA/I	CS6^+^ reference ETEC	142	124	2	123	1,379
CS6	CFA/I^+^ reference ETEC	87	179	4	42	1,227
Phylogroup A CFA/I	Phylogroup B1 CFA/I	119	22	100	422	2,091
Phylogroup B1 CFA/I	Phylogroup A CFA/I	22	119	137	478	1,142
CFA/I ETEC lineage L3	Other CFA/I	21	121	119	243	665
CFA/I ETEC lineage L6	Other CFA/I	105	37	146	260	1,201
CFA/I ETEC lineage L15	Other CFA/I	14	128	157	270	420
Phylogroup A CS6	Phylogroup B1 CS6	16	71	22	361	1,407
Phylogroup B1 CS6	Phylogroup A CS6	71	16	58	307	2,246
CS6 ETEC lineage L4	Other CS6	9	78	136	166	380
CS6 ETEC lineage L5	Other CS6	53	34	50	139	982
CS6 ETEC lineage L8	Other CS6	16	71	104	267	459

a The *de novo* LS-BSR analysis included 142 CFA/I genomes (ST^+^, LT^−^, and CFA/I^+^), 87 CS6 genomes (ST^+^, LT^−^, and CS6^+^), and 37 reference ETEC genomes (LT^+^ and/or ST^+^ but not containing CFA/I or CS6). There were 3,567 gene clusters identified with significant similarity (BSR of ≥0.9) in all 266 ETEC genomes analyzed (CS6, CFA/I, and reference ETEC).

b Data represent numbers of gene clusters that were present in all genomes, ≥50% of the genomes, or ≥1 of the genomes of group 1 (BSR of ≥0.9) and absent from all of the genomes of group 2 (BSR of <0.4).

10.1128/mSystems.00329-18.6 TABLE S2 Gene clusters in CFA/I ETEC isolates that were absent from other reference isolates. Download Table S2, XLSX file, 0.7 MB. Copyright © 2019 Hazen et al. 2019 Hazen et al. This content is distributed under the terms of the Creative Commons Attribution 4.0 International license.

The number of genes that were shared among the CFA/I or CS6 genomes increased for genomes of the same phylogroup or lineage, demonstrating that there were a greater number of phylogroup and lineage-specific genes than genes associated with CF type (Table 1). The number of lineage-specific genes that were identified in all genomes of one lineage and absent from other genomes of the same CF type ranged from 50 to 136 among the three dominant CS6 lineages (L4, L5, and L8), and from 60 to 78 among the dominant CFA/I lineages (L3, L6, and L15) (Table 1). These findings demonstrate that certain lineages of CFA/I or CS6 ETEC had a greater number of lineage-specific genes. The genes that were conserved at the phylogroup level among the CFA/I or CS6 ETEC isolates included genes associated with a type II secretion system (T2SS) and genes with predicted functions involved in metabolism, while the genes that were unique to particular lineages included genes associated with metabolism and also mobile-element-associated genes, especially phage-associated genes (Table S2).

### CF-associated distribution of toxins and other virulence genes among the CFA/I and CS6 ETEC isolates.

*In silico* detection of the ST and LT genes in each of the PCR-based presumptive ST-only ETEC genomes verified that 89% (239/269) of the genomes had only the ST gene and not the LT genes, whereas four genomes had the genes for both LT and STh (Table S1). Although all of the ETEC isolates included in this study were PCR positive (PCR^+^) for the ST gene, 9% (26/269) of the isolates were missing this gene from their genome assemblies (Table S1). There were 18 presumptive CS6 ETEC genomes that were missing the genes that encode CS6, with 61% (11/18) of these genomes also missing ST, and 19 genomes were missing the genes that encode CFA/I, with 74% (14/19) of these genomes also missing ST (Table S1). The gene encoding ST and the CS6 and CFA/I genes typically occur on plasmids that in some instances have demonstrated instability (20–22, 25–27). Thus, it is possible that these ETEC isolates had previously carried an ST-encoding and/or CS6- or CFA/I-encoding plasmid that was lost during laboratory passage. Identification of the previously described ST gene alleles (28) in each of the ETEC genomes demonstrated that the *estA2* allele was present in all but three of the CFA/I ETEC isolates whereas the CS6 ETEC genomes carried *estA3*, *estA4*, *estA5*, or *estA7* alleles (see Fig. S1 in the supplemental material). Interestingly, the *estA2* allele was also identified in five CS6 ETEC isolates, and all of these ETEC isolates were present in an undesignated ETEC lineage (Fig. 1; see also Fig. S1).

10.1128/mSystems.00329-18.1 FIG S1 Phylogenetic analysis of STh and STp. Nucleotide sequences of STh and STp genes were aligned with *estA1* to *estA7* reference sequences using ClustalW, and a phylogeny was constructed using the maximum likelihood method with the Kimura 2-parameter model and 1,000 bootstraps using MEGA7 (15). The clades and isolate labels are colored according to the *estA* allele (see inset legend). The presence of genes encoding CS6 and the presence of genes encoding CFA/I are indicated by symbols adjacent to each genome label (see inset figure legend). The filled symbols indicate the CF types that were identified by an initial PCR screen and also in the genome assembly. An open symbol indicates that the CF types were detected by PCR but were absent from the genome assembly. The tree scale indicates a distance of 0.1 nucleotide changes per site. Bootstrap values of ≥50 are indicated by gray circles. Download FIG S1, PDF file, 0.08 MB. Copyright © 2019 Hazen et al. 2019 Hazen et al. This content is distributed under the terms of the Creative Commons Attribution 4.0 International license.

**FIG 3:**
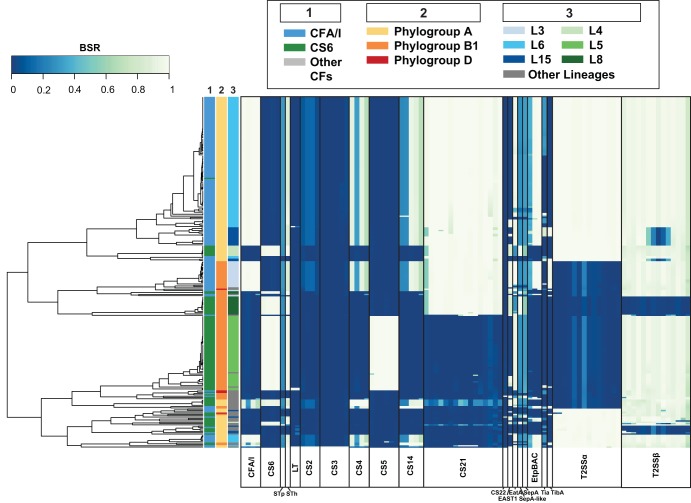
*In silico* detection of ETEC virulence genes. ETEC virulence genes that were previously described (60) were detected in each of the ST-only ETEC genomes using BLASTN LS-BSR. Each column represents a gene encoding the specified virulence factors indicated by labels at the bottom of the heat map. Colors of the heat map indicate virulence genes that were detected with significant similarity (light green) or with divergent similarity (blue-green) or were absent (dark blue) in each of the genomes analyzed. Rows represent individual genomes that are categorized on the left by three columns as follows: column 1, colonization factor content; column 2, phylogroup; column 3, ETEC phylogenomic lineage (see inset legend for color designations).

There were two or more CFs identified in 88% (236/269) of the genomes, with 90% (146/162) of the CFA/I isolates and 84% (90/107) of the CS6 isolates carrying additional CFs (Table S1). Interestingly, CS21 (29, 30) was identified in 88% (142/162) of the CFA/I genomes, compared with only 29% (31/107) of the CS6 genomes (*P* value of <0.001) (Table 2). The genes encoding CS5 were identified in 53% (57/107) of the CS6 genomes and in none of the CFA/I genomes (*P* value of <0.001) (Table 2). Minor CFs (CS2, CS3, CS4, CS14, and CS22) were identified in ≤5 of the CFA/I and CS6 ETEC genomes (Table 2). Additional virulence genes were also detected that encode predicted proteins involved in adhesion to the host surface, including genes encoding the autotransporters EatA (31), TibA (32), and SepA from *Shigella* (33) and a *sepA*-like gene that had 83% nucleotide identity to *sepA* from *Shigella* compared to 75% nucleotide identity to *eatA* (Table 2). The EatA gene was identified in 85% (137/162) of the CFA/I genomes compared with 62% (66/107) of the CS6 genomes (*P* value of <0.001) (Table 2). TibA was identified in 21% (34/162) of the CFA/I ETEC genomes but in only 4% (4/107) of the CS6 ETEC genomes (*P* value of <0.001) (Table 2). The adhesin EtpA (34) was identified in 89% (144/169) of the CFA/I ETEC genomes compared to only 7% (8/107) of the CS6 ETEC genomes (Table 2). Additional gene regions that may contribute to virulence, including a type II secretion system (T2SS), were detected in the CFA/I and CS6 ETEC genomes (Table 2) (Fig. 3; see also Text S1 in the supplemental material).

**TABLE 2:** Summary of ETEC virulence factor content in the CS6- and CFA/I ST-only ETEC genomes

Virulence factor	GenBank accession no. or source^*a*^	No. (%) of genomes^*b*^			*P* value^*c*^
		All CFA/I and CS6	CFA/I	CS6	
Toxins					
STIa (STp)	YP_003294006.1	1 (<1)	0 (0)	1 (1)	NS
STIb (STh)	WP_023485648.1	242 (90)	147 (91)	95 (89)	NS
LT-I	YP_003293996.1–YP_003293997.1	4 (1)	2 (1)	2 (2)	NS
EAST1	AAD43571.1	80 (30)	37 (23)	43 (40)	0.0036

Colonization factors and adhesins					
CFA/I	CBJ04486.1–CBJ04489.1	144 (54)	143 (88)	1 (1)	<0.001
CS6	AAC45093.1–AAC45096.1	89 (33)	0 (0)	89 (83)	<0.001
CS2	CAA87760.1–CAA87763.1	1 (<1)	1 (<1)	0 (0)	NS
CS3	CAA34815.1–CAA34820.1	1 (<1)	1 (<1)	0 (0)	NS
CS4	AAK97134.1–AAK97137.1	5 (2)	0 (0)	5 (5)	0.0094
CS5	CAA11821.1–CAA11825.1	57 (21)	0 (0)	57 (53)	<0.001
CS14	AAQ20104.1–AAQ20108.1	5 (2)	3 (2)	2 (2)	NS
CS21	ABU50035.1–ABU50050.1	167 (62)	142 (88)	31 (29)	<0.001
CS22	AAD30557.1	1 (<1)	1 (<1)	0 (0)	NS
EtpA (EtpBAC)	AAX13508.1–AAX13510.1	152 (57)	144 (89)	8 (7)	<0.001
EatA	AAO17297.1	203 (75)	137 (85)	66 (62)	<0.001
SepA-like	This study	30 (11)	2 (1)	28 (26)	<0.001
SepA	AAL72309.1	4 (1)	2 (1)	2 (2)	NS
Tia	AAB06592.1	15 (6)	13 (8)	2 (2)	NS
TibA	CBJ01643.1	38 (14)	34 (21)	4 (4)	<0.001

Other					
T2SSα	CBJ03075.1–CBJ03088.1	156 (58)	131 (81)	25 (23)	<0.001
T2SSβ	CBJ02728.1–CBJ02741.1	246 (91)	159 (98)	87 (81)	<0.001

a The GenBank accession numbers are indicated for the protein sequences.

b Data represent numbers of genomes that had each virulence factor with a BSR of ≥0.8, identified using both TBLASTN and BLASTN. The total numbers of genomes analyzed were as follows: all CFA/I and CS6, *n* = 269; CFA/I, *n* = 162; CS6, *n* = 107. The percentages of the genomes are indicated in parentheses.

c *P* values were generated by comparing the number of CFA/I genomes to the number of CS6 genomes that had each virulence factor by the chi square test (or Fisher's exact test when present in ≤5 genomes) using R v.3.4.1. NS, not significant (*P* value of >0.05).

10.1128/mSystems.00329-18.4 TEXT S1 Supplemental results and references. Download Text S1, DOCX file, 0.1 MB. Copyright © 2019 Hazen et al. 2019 Hazen et al. This content is distributed under the terms of the Creative Commons Attribution 4.0 International license.

### Comparison of complete genomes reveals geographic variation among CFA/I and CS6 ETEC isolates.

Based on epidemiological data and laboratory-based characterizations, we selected 26 ST-only ETEC isolates for complete genome sequencing to provide additional insight into the diversity of plasmids and other genomic regions in these isolates, as well as to further inform the selection of candidate challenge strains for use in human volunteer challenges (Table S3). These ETEC isolates met the following selection criteria making them potential candidates as future challenge strains: (i) they were associated with moderate to severe diarrhea in humans; (ii) they encoded only ST and not LT; (iii) they encoded CS6 or CFA/I; (iv) they were not of serogroup O39, O71, O78, or O141, which are represented by current whole-cell ETEC vaccine candidates which are in advanced clinical development (35–38); and (v) they were susceptible to a panel of eight commonly used antibiotics (azithromycin, ampicillin/sulbactam, cefazolin, ceftriaxone, ciprofloxacin, levofloxacin, tetracycline, and trimethoprim-sulfamethoxazole) (Tables S3 and S4). The CFA/I and CS6 isolates that qualified for additional genome sequencing included six CFA/I and 20 CS6 isolates, which were isolated between 1974 and 2012 in eight different countries (Table S3). These isolates represented 11 MLST sequence types and 11 serotypes and belonged to seven of the ETEC phylogenomic lineages (Table S3). Western blot analysis verified the production of CFA/I and CS6 by these isolates, while the hemagglutination assay verified the activity of CFA/I (Table S3).

10.1128/mSystems.00329-18.7 TABLE S3 Characteristics of the ST-only ETEC genomes selected for complete genome sequencing. Download Table S3, XLSX file, 0.06 MB. Copyright © 2019 Hazen et al. 2019 Hazen et al. This content is distributed under the terms of the Creative Commons Attribution 4.0 International license.

10.1128/mSystems.00329-18.8 TABLE S4 Antimicrobial susceptibilities of the CS6- and CFA/I ST-only ETEC isolates. Download Table S4, XLSX file, 0.09 MB. Copyright © 2019 Hazen et al. 2019 Hazen et al. This content is distributed under the terms of the Creative Commons Attribution 4.0 International license.

**FIG 4:**
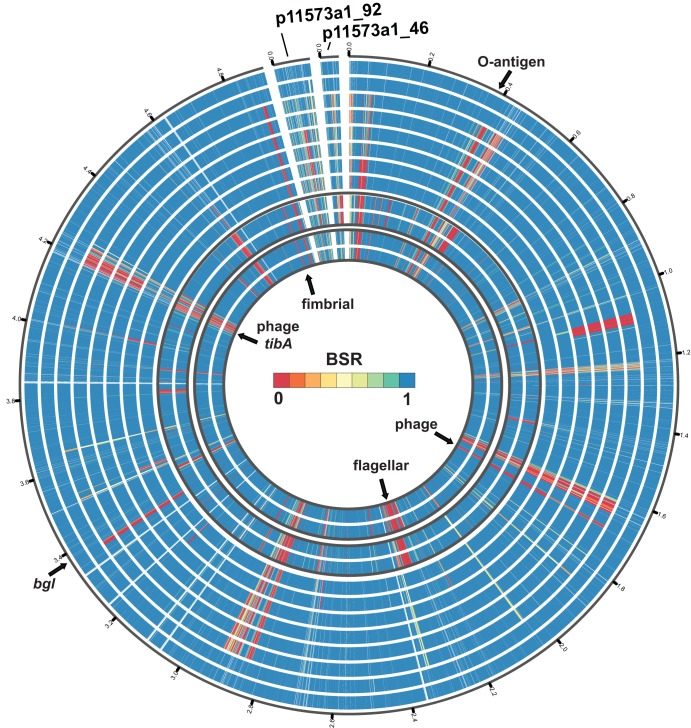
Sequence comparison of the genome of lineage L15 ETEC isolate 11573a-1 to the genomes of other representative CFA/I ETEC isolates. Protein-coding genes located on the chromosome and plasmids of the CFA/I ETEC isolate 11573a-1 were identified in the genomes of other CFA/I ETEC isolates using BLASTN LS-BSR (60). The data tracks are numbered 1 to 12 from the outer ring (ring 1) to the inner ring (ring 12). The outer eight tracks indicate the presence (blue), divergent similarity (yellow), and absence (red) of genes in eight ETEC genomes from lineage L15 as follows: 10754a-1 (track 1), 10802a (track 2), 710903 (track 3), 700384 (track 4), 600609 (track 5), 500469 (track 6), 320116 (track 7), and 300252 (track 8). Tracks 9 and 10 contain genomes of lineage L3 as follows: 310142 (track 9) and 620828 (track 10). Tracks 11 and 12 contain genomes of lineage L6 as follows: 102625 (track 11) and A44 (track 12).

Comparison of a representative complete genome from each of the three dominant CS6 phylogenomic lineages and the three dominant CFA/I lineages demonstrated that these genomes have plasmid and chromosomal regions that exhibit lineage and geographic specificity (Fig. 4; see also Fig. S2A to E and Table S5A to F). There were multiple genome regions identified in CFA/I isolate 11573 a-1 from lineage L15 that were absent from the genomes of isolates from other CFA/I lineages and in some cases were also missing from isolates belonging to the same lineage that were from different geographic locations (Fig. 4; see also Table S5C). One of the genome regions that was present in the lineage L15 genomes from Chile (11573 a-1, 10754 a-1, and 10802 a) but absent or had divergent similarity in the representative lineage L15 genomes from Mozambique (300252 and 320116), India (500469), Bangladesh (600609), and Pakistan (700384 and 710903) consisted of genes involved in O-antigen biosynthesis (EC11573a1_358 to EC11573a1_370) (Table S5C). The three lineage L15 CFA/I ETEC isolates from Chile (11573 a-1, 10754 a-1, and 10802 a) had *in silico*-predicted serotype O49:H12, while each of the L15 genomes from other geographic locations (300252, 320116, 500469, 600609, 700384, 710903) had *in silico*-predicted serotype O128ac:H12 (Table S1). Although these isolates were all sequence type ST10, they have likely undergone recombination within their O-antigen biosynthesis regions. The genome of ETEC isolate 11573 a-1 also contained lineage-specific regions that were conserved among the L15 genomes but absent from the representative CFA/I genomes of L3 and L6, which included putative genes involved in flagellum biosynthesis (EC11573a1_2179 to EC11573a1_2218) (Fig. 4; see also Table S5C).

10.1128/mSystems.00329-18.2 FIG S2 (A) Comparison of the genome of lineage L3 CFA/I ETEC isolate 504838 to the genomes of other CFA/I ETEC isolates. Protein-coding genes located on the chromosome and plasmids of CFA/I ETEC isolate 504838 were detected in selected CFA/I ETEC genomes using BLASTN LS-BSR (16). The outer eight tracks indicate the presence (blue), divergent similarity (yellow), and absence (red) of genes in eight additional ETEC genomes from lineage L3 as follows: 310142 (track 1), 310202 (track 2), 401963 (track 3), 402411 (track 4), 521034 (track 5), 621178 (track 6), 620828 (track 7), and 710663 (track 8). Tracks 9 and 10 contain genomes of lineage L6 as follows: 102625 (track 9) and A44 (track 10). Tracks 11 and 12 contain genomes of lineages L15 as follows: 11573a-1 (track 11) and 710903 (track 12). (B) Comparison of the genome of lineage L6 CFA/I ETEC isolate 504239 to the genomes of other CFA/I ETEC isolates. Protein-coding genes located on the chromosome and plasmids of CFA/I ETEC isolate 504239 were detected in selected CFA/I ETEC genomes using BLASTN LS-BSR (16). The outer eight tracks indicate the presence (blue), divergent similarity (yellow), and absence (red) of genes in eight additional ETEC genomes from lineage L6 as follows: A44 (track 1), 102625 (track 2), 210088 (track 3), 302824 (track 4), 410529 (track 5), 401981 (track 6), 504866 (track 7), and 520657 (track 8). Tracks 9 and 10 contain genomes of lineage L3 as follows: 310142 (track 9) and 620828 (track 10). Tracks 11 and 12 contain genomes of lineages L15 as follows: 11573a-1 (track 11) and 710903 (track 12). (C) Comparison of the genome of lineage L4 CS6 ETEC isolate 203740 to the genomes of other CS6 ETEC isolates. Protein-coding genes located on the chromosome and plasmids of the CS6 ETEC isolate 203740 were detected in selected CS6 ETEC genomes using BLASTN LS-BSR (16). The outer eight tracks indicate the presence (blue), divergent similarity (yellow), and absence (red) of genes in eight additional ETEC genomes from lineage L4 as follows: 120158 (track 1), 204044 (track 2), 603744 (track 3), 603766 (track 4), 700508 (track 5), 710421 (track 6), 710667 (track 7), and 720828 (track 8). Tracks 9 and 10 contain genomes of lineage L5 as follows: 103605 (track 9) and 120899 (track 10). Tracks 11 and 12 contain genomes of lineages L8 as follows: 500465 (track 11) and 503025 (track 12). (D) Comparison of the genome of lineage L5 CS6 ETEC isolate 120899 to the genomes of other CS6 ETEC isolates. Protein-coding genes located on the chromosome and plasmids of CS6 ETEC isolate 120899 were detected in selected CS6 ETEC genomes using BLASTN LS-BSR (16). The outer eight tracks indicate the presence (blue), divergent similarity (yellow), and absence (red) of genes in eight additional ETEC genomes from lineage L5 as follows: 103605 (track 1), 202556 (track 2), 310003 (track 3), 403395 (track 4), 503606 (track 5), 602354 (track 6), 703595 (track 7), and 702551 (track 8). Tracks 9 and 10 contain genomes of lineage L4 as follows: 120158 (track 9) and 720828 (track 10). Tracks 11 and 12 contain genomes of lineages L8 as follows: 403030 (track 11) and 700308 (track 12). (E) Comparison of the genome of lineage L8 CS6 ETEC isolate 503025 to the genomes of other CS6 ETEC isolates. Protein-coding genes located on the chromosome and plasmids of CS6 ETEC isolate 503025 were detected in selected CS6 ETEC genomes using BLASTN LS-BSR (16). The outer eight tracks indicate the presence (blue), divergent similarity (yellow), and absence (red) of genes in eight additional ETEC genomes from lineage L8 as follows: 201399 (track 1), 403030 (track 2), 503829 (track 3), 500465 (track 4), 503440 (track 5), 702193 (track 6), 702915 (track 7), and 700308 (track 8). Tracks 9 and 10 contain genomes of lineage L4 as follows: 120158 (track 9) and 203740 (track 10). Tracks 11 and 12 contain genomes of lineages L5 as follows: 202789 (track 11) and 703595 (track 12). Download FIG S2, PDF file, 19.7 MB. Copyright © 2019 Hazen et al. 2019 Hazen et al. This content is distributed under the terms of the Creative Commons Attribution 4.0 International license.

10.1128/mSystems.00329-18.9 TABLE S5 Detection of protein-coding genes of CFA/I ETEC reference isolates among the other ETEC genomes analyzed. Download Table S5, XLS file, 18.5 MB. Copyright © 2019 Hazen et al. 2019 Hazen et al. This content is distributed under the terms of the Creative Commons Attribution 4.0 International license.

### Distribution of a conserved CFA/I-encoding plasmid and multiple unique CS6-encoding plasmids.

**FIG 5:**
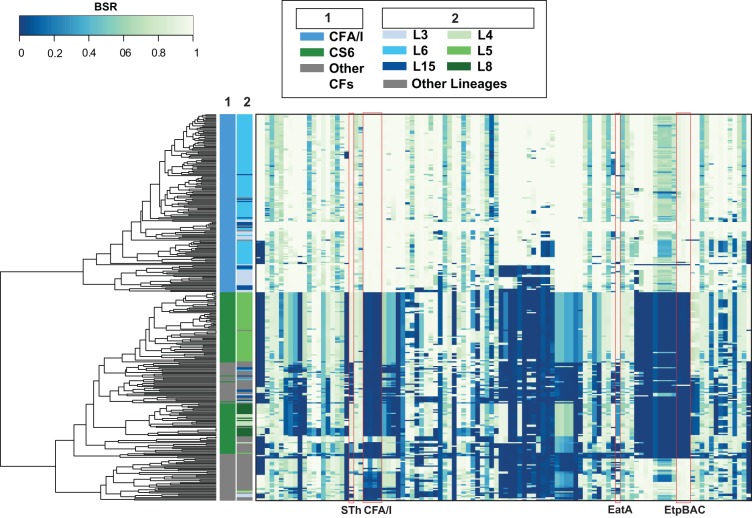
*In silico* detection of an STh and a CFA/I-encoding plasmid. The predicted protein-coding genes of STh, CFA/I, EatA, and EtpBAC-encoding plasmid p11573a1_92 were detected in all of the ETEC genomes analyzed using BLASTN LS-BSR (60). The rows represent individual genomes that are labeled on the left side by their colonization factor content (column 1) and by their phylogenomic lineage (column 2) (see inset figure legend for colors). Each column represents a different protein-coding gene of plasmid p11573a1_92. The virulence factors are indicated by a red box.

The CFA/I and STh genes were colocated on the same plasmid in all six of the complete CFA/I genomes (Table S3). These plasmids ranged in size from 88.8 to 101.6 kb, had the IncFII(AY458016) replicon, and also carried the *eatA* gene (Table S3), which encodes the serine protease autotransporter EatA (31). *In silico* detection of STh, CFA/I, and EatA plasmid p11573a1_92 from ETEC isolate 11573 a-1 demonstrated that this plasmid was highly conserved among all of the CFA/I ETEC isolates examined in this study (Fig. 5). The CFA/I ETEC genomes also contained an IncFIB plasmid that ranged in size from 46.6 to 155.8 kb and carried genes encoding CS21 (29, 30) (Table S3). Interestingly, the CS21 genes were identified in 88% (142/162) of the CFA/I genomes compared to only 29% (31/107) of the CS6 genomes (*P* value of <0.001) (Table 2). The genes of CS21-encoding plasmid p11573a1_46 from ETEC isolate 11573 a-1 were identified in nearly all of the L6 and L15 CFA/I ETEC genomes; however, a region of the CS21 plasmid with approximately 17 genes, encoding mostly hypothetical proteins, was absent from the L3 CFA/I genomes and also from the CS6 genomes that encode CS21 (Fig. S3A).

10.1128/mSystems.00329-18.3 FIG S3 Distribution of CS6 and CFA/I plasmids. Data represent results of *in silico* detection of (A) the CS21 plasmid, p11573_46, of CFA/I ETEC isolate 11573 a-1; of (B) STh, CS5, CS6, and the EatA plasmid, p120899_146, of CS6 ETEC isolate 120899; of (C) STh, CS6, SepA, and the EAST1 plasmid, p500465_77, of CS6 ETEC isolate 500465; of (D) STp, CS6, and the CS21 plasmid, p214_4_132, of CS6 ETEC isolate 214-4; of (E) STh, CS4, and CS21 plasmid p503046_85; and of (F) CS6, EatA, and EAST1 plasmid p503046_80 of CS6 ETEC isolate 503046. The predicted protein-coding genes of each plasmid were identified in all of the ETEC genomes analyzed using BLASTN LS-BSR (16). The rows represent individual genomes labeled on the left side by their colonization factor content (column 1) and by their phylogenomic lineage (column 2) (see inset figure legend for colors). Each column represents a different protein-coding gene of the reference plasmid selected for comparison. The virulence factors are indicated by a red box. Download FIG S3, PDF file, 2.8 MB. Copyright © 2019 Hazen et al. 2019 Hazen et al. This content is distributed under the terms of the Creative Commons Attribution 4.0 International license.

In contrast to the conserved CFA/I^+^ STh plasmid that was identified, three unique ST^+^ CS6 plasmids were identified among the CS6 ETEC genomes (Table S3; see also Fig. S3B to D). A plasmid encoding both ST and CS6 was identified in 70% (14/20) of the complete CS6 ETEC genomes, while four of the CS6 ETEC isolates (503046, 503458, 510016, and 520873) carried STh and CS6 on separate plasmids, and the two remaining PCR-verified CS6 ETEC isolates (600468 and 720632) were missing the CS6 genes from their complete genome assemblies, but each had an STh-encoding plasmid (Table S3). The three unique plasmids that encoded both ST and CS6 also exhibited lineage specificity, with one ST^+^ CS6 plasmid detected only in the lineage L5 CS6 ETEC genome (Fig. S3B), and a second ST^+^ CS6 plasmid in the CS6 ETEC genomes of lineages L4 and L8 (Fig. S3C). The third ST^+^ CS6 plasmid encoded STp rather than STh and was identified only in ETEC isolate 214-4 (Fig. S3D). Interestingly, the four complete genomes that had STh and CS6 genes on two separate plasmids were identified in a single undesignated lineage of phylogroup A (Fig. 1; see also Table S3). *In silico* detection of the STh (p503046_85) and CS6 (p503046_80) plasmids demonstrated that both of these plasmids were present in all five of the ETEC genomes of this lineage (503046, 702582, 503458, 520873, and 510016) (Fig. S3E and F). These plasmids were not present in any of the other ETEC genomes analyzed (Fig. S3E and F), demonstrating that two unique plasmids were involved in the acquisition of STh and CS6 by ETEC isolates of this novel ETEC lineage. Identification of the ST genes among the genomes of this lineage demonstrated that the ST plasmid of these CS6 ETEC genomes contained the *estA2* allele, which is typically carried by the CFA/I ETEC (Fig. S1).

## DISCUSSION

Previous studies, including the case-control GEMS, demonstrated that ST-only ETEC strains are among the leading causes of severe diarrheal illness among children and are more often associated with severe illness than ETEC strains that encode only LT (2, 7, 9, 67). Thus, in the current study we investigated whether there are genomic or phenotypic differences among the dominant CF types (CS6 and CFA/I) of the ST-only ETEC strains. Phylogenomic analysis demonstrated that a majority of the CFA/I ETEC and CS6 ETEC strains occur in six distinct lineages, although they were identified in up to 13 previously described ETEC lineages in all, as well as additional undefined lineages, revealing that genomically diverse E. coli strains have acquired the genes encoding ST and either CFA/I or CS6. Previous comparative genomics studies have demonstrated an association of particular toxins and CFs with different lineages of ETEC (10, 20, 21, 39–41). Similarly, we observed an association of ST and certain CFs with the previously designated ETEC lineages; however, we also determined that a number of noncanonical ETEC virulence factors, including autotransporters and secretion systems, exhibited lineage specificity. In some cases, the noncanonical virulence genes exhibited a greater association with their dominant CF type (CFA/I or CS6) than with their lineage, suggesting that certain noncanonical virulence genes are colocated with the CF genes on plasmids or other mobile elements. Interestingly, gene-based comparisons of the CFA/I and CS6 ETEC isolates identified phylogroup and lineage-specific genes but also demonstrated there was geographic specificity in the genome content among isolates belonging to the same lineage. Many of the variable regions in the CFA/I and CS6 ETEC genomes contained genes associated with phage or transposable elements, highlighting the role of mobile elements in the ongoing diversification of the CFA/I and CS6 ETEC strains (and most likely all ETEC strains).

By generating complete genome sequences of selected CFA/I and CS6 ETEC isolates, we were also able to describe plasmids that encode ST and CS6 or CFA/I. Interestingly, the STh- and CFA/I-encoding plasmids were highly conserved among the CFA/I ETEC isolates analyzed in this study, suggesting that the CFA/I ST-only ETEC lineages most likely arose by the acquisition of this conserved virulence plasmid by multiple genomically diverse E. coli lineages. In contrast, the completed CS6 ETEC genomes have several unique ST and/or CS6-encoding plasmids, which have been acquired by multiple genomically diverse E. coli lineages. Interestingly, functional characterization demonstrated that CS6 ETEC isolates of different lineages that have unique virulence plasmids also exhibited significant differences in their ST production. Further investigation is necessary to determine whether plasmid or chromosomal genes are contributing to differences in ST production and, if so, whether this results in differences in illness severity associated with these ST-only ETEC isolates.

In summary, our findings demonstrate that while the majority of the CFA/I ST-only ETEC and CS6 ST-only ETEC analyzed were present in a limited number of dominant lineages, the genes encoding ST, CFA/I, and CS6 had been acquired by genomically diverse ETEC by the dissemination of a highly conserved CFA/I-encoding plasmid and several different versions of a CS6-encoding plasmid. Furthermore, variation was identified in the genome content of the CFA/I ETEC and CS6 ETEC isolates that was associated with geographic location of isolation, phylogroup, or lineage, demonstrating that selected populations of ST-only ETEC strains have undergone additional diversification following the acquisition of the ST and CF genes. There is currently no approved vaccine for disease caused by ST-only ETEC, or by any ETEC strain for that matter, and as such, the current report provides functional verification of ST and CF production, antimicrobial susceptibility testing data, and an in-depth genomic characterization of isolates that could serve as representatives of CFA/I- or CS6-encoding ST-only ETEC strains for future studies of ETEC pathogenesis, vaccine studies, and/or clinical trials. These isolates will be further functionally investigated for differences in their gene content that influences ST production and are planned to be developed as potential challenge isolates for use in evaluating future vaccine candidates.

## MATERIALS AND METHODS

### ETEC isolates.

The E. coli isolates from the GEMS collection and ETEC isolates from diarrheal cases in Chile (D. A. Rasko et al., unpublished data) (42, 43), were PCR screened for the presence of LT and ST and colonization factors as previously described (44). ETEC isolates 214-4 (13) (STp, CS6), TW11681 (41) (STh, CFA/I, CS21), and TW10590 (45) (STh, CFA/I, CS21) were included as archetypal isolates that encode ST and CS6 or CFA/I.

### Antimicrobial susceptibilities.

The Kirby-Bauer disk diffusion method was used to determine the susceptibility of the 269 ETEC isolates examined in this study against 15 µg azithromycin, 10 µg ampicillin/10 µg sulbactam, 30 µg cefazolin, 30 µg ceftriaxone, 5 µg ciprofloxacin, 5 µg levofloxacin, 30 µg tetracycline, and 1.25 µg trimethoprim/23.75 µg sulfamethoxazole (46).

### Serogroups.

The O antigen was determined as described previously by Guinée et al. (47) using antisera that identify O antigen serogroups O1 to O185. Isolates that did not react with O antisera were classified as nontypeable (ONT). All antisera were obtained and adsorbed with the corresponding cross-reacting antigens to remove nonspecific agglutinins.

### Production and activity of CFA/I and CS6.

Whole-cell lysates were prepared from ETEC isolates grown on CFA agar (CFA/I ETEC) or in lysogeny broth (LB) (CS6 ETEC), normalized according to optical density at 600 nm (OD_600_), and mixed 1:1 with 2× Laemmli buffer. Samples were electrophoresed by 15% SDS-PAGE, and proteins were transferred to polyvinylidene difluoride (PVDF) membranes (Millipore Corp., Bedford, MA). The membranes were probed with rabbit anti-CFA/I or anti-CS6 antibody (Rockland, Limerick, PA). Western immunoblots were developed using an Odyssey system (Li-Cor Biosciences, Lincoln, NE). Positive controls included purified protein samples of CFA/I or CS6 (BEI Resources, Manassas, VA).

The ability of CFA/I-expressing ETEC to hemagglutinate (HA) human type A red blood cells (RBC) was assessed. Duplicate samples of ETEC isolates grown on CFA agar were resuspended to an OD_600_ of 2.0 and serially diluted 2-fold in phosphate-buffered saline (PBS) in a 96-well plate. An equal volume of washed human type A RBC was added to each well. Equal volumes of 0.1 M d-(+) mannose–0.15 M NaCl were added to all wells. Plates were incubated for 2 h at 4°C. The hemagglutinin (HA) titer of each isolate was read as the dilution at which the RBC pellet did not form at the bottom of the well.

### ST production.

Selected ETEC isolates were grown overnight in LB and were used to inoculate chemically defined 4AA medium at a 1:100 dilution and were incubated overnight at 37°C and 250 rpm. 4AA medium is a chemically defined medium that has been used successfully for ST expression and subsequent purification (23, 48). The following morning, the culture OD_600_ was recorded, 1 ml of each culture was centrifuged at 13,000 rpm for 10 min, and 800 µl of supernatant was immediately divided into aliquots, placed into 2.0-ml glass screw vials, and frozen at −20°C until assayed for ST activity via the cGMP assay. Human T84 colonic epithelial cells were purchased from the American Type Culture Collection (ATCC) (catalog no. CCL-248) and were cultured in ATCC’s 1:1 Dulbecco’s modified Eagle’s medium and Ham’s nutrient mixture F-12 (DMEM–F-12; Gibco catalog no. 11320033) containing 2.5 mM l-glutamine, 15 mM HEPES, and 0.5 mM sodium pyruvate and supplemented with 5% fetal bovine serum (FBS). All cell cultures were supplemented with antibiotic-antimycotic (Gibco). Confluent T84 cells were harvested from T-75 culture flasks using 0.25% trypsin and resuspended in DMEM–F-12 medium. T84 cells were seeded into 24-well, flat-bottom cell culture plates (Corning Costar, Cambridge, MA) at a density of 5 × 10^5^ cells per well and grown to confluence. Intracellular cGMP levels were determined as previously described (24). The amount of ST produced by the ETEC isolates was calculated relative to the amount of cGMP produced by the 10 ng of purified ST-positive control. Statistical differences in the mean levels of ST production by ETEC isolates associated with the colonization factor type (CFA/I or CS6) or from different lineages were determined with R v.3.4.1 using the F test of variance and the two-sample *t* test.

### Genome sequencing and assembly.

Genomic DNA of each ETEC isolate was extracted from overnight cultures using a Sigma GenElute bacterial genomic DNA kit (Sigma-Aldrich; St. Louis, MO). The genomes were sequenced using paired-end 500-bp insertion libraries and an Illumina HiSeq 4000 system. The 150-bp Illumina reads were assembled using SPAdes v.3.7.1 (49), and the final assemblies were filtered to contain only contigs that were ≥500 bp in length and had ≥5× k-mer coverage. The assembly metrics are provided in Table S1 in the supplemental material. Additional long-read genome sequencing was performed on a Pacific Biosciences RS II platform (PacBio) as previously described (50, 51). The characteristics of the complete assemblies are listed in Table S3.

### *In silico* multilocus sequence typing, serotyping, and detection of antibiotic resistance genes.

The seven genomically conserved housekeeping loci (*adk*, *gyrB*, *fumC*, *icd*, *mdh*, *purA*, and *recA*) of the multilocus sequence typing (MLST) scheme previously developed by Wirth et al. (52) were identified in each of the genomes listed in Table S1 as previously described (51). These genes are used to examine the population structures of the compared E. coli isolates. The serotypes were predicted using Serotype Finder v. 1.1 (https://cge.cbs.dtu.dk/services/SerotypeFinder/) (53). Antibiotic resistance genes were identified in each of the ETEC genomes using resistance gene identifier (RGI) v.3.2.0 of the comprehensive antibiotic resistance database (CARD) (54) as previously described (50, 51).

### Phylogenomic analysis.

The 269 CFA/I and CS6 ETEC genomes analyzed in this study were compared with 61 previously sequenced ETEC reference genomes (Table S1) and 31 diverse E. coli and *Shigella* genomes (55) using a single nucleotide polymorphism (SNP)-based approach as previously described (56, 57). There were 204,335 conserved SNP sites among these genomes relative to the reference E. coli IAI39 genome (GenBank accession no. NC_011750.1). The concatenated SNP sites were used to infer a maximum likelihood phylogeny with RAxML v7.2.8 (58), using the GTR model of nucleotide substitution, the GAMMA model of rate heterogeneity, and 100 bootstrap replicates. The phylogeny was labeled using interactive Tree Of Life software (iTOL v.3) (59).

### Gene-based comparisons.

Differences in gene content among the CS6 ETEC and CFA/I ETEC isolates were identified using BLASTN large-scale BLAST score ratio (LS-BSR) analysis as previously described (60, 61). The protein-coding genes of each genome were assigned to gene clusters with ≥90% nucleotide identity and ≥90% alignment length using CD-HIT v. 4.6.7 (62) (see Data Set S1 in the supplemental material). Gene clusters identified with a BSR of ≥0.9 were considered to represent significant similarity, while gene clusters with a BSR of <0.4 were considered absent.

10.1128/mSystems.00329-18.10 DATA SET S1 Nucleotide sequence of the clusters in fasta format. Download Data Set S1, TXT file, 17.4 MB. Copyright © 2019 Hazen et al. 2019 Hazen et al. This content is distributed under the terms of the Creative Commons Attribution 4.0 International license.

### *In silico* detection of E. coli virulence genes and plasmids.

E. coli and *Shigella* virulence genes were identified in the ETEC genomes also using BLASTN LS-BSR as previously described (60, 61). The association of virulence genes among the CFA/I ETEC and CS6 ETEC genomes was analyzed for statistical significance using Pearson’s chi-square test with Yates’ continuity correction or Fisher’s exact test using R v.3.4.1. The clustered heat maps were generated using the heatmap2 function of gplots v. 3.0.1 in R v.3.3.2 and the complete linkage method with Euclidean distance estimation. Plasmid incompatibility types in the PlasmidFinder v.1.3 database (63) were identified in each of the ETEC genomes using BLASTN LS-BSR (60, 61). Plasmids in each of the complete genomes were annotated using an in-house annotation pipeline (64, 65). The predicted protein-coding genes of selected plasmids were detected in each of the ETEC genomes using BLASTN LS-BSR and were visualized as a clustered heat map as described above.

The sequences of the ST genes from each ETEC genome were compared with previously described *estA* reference sequences (28). The *estA* nucleotide sequences were aligned using ClustalW, and a phylogeny was constructed using the maximum likelihood method with the Kimura 2-parameter model and 1,000 bootstraps using MEGA7 (66), and the results were labeled using iTOL (59).

### Data availability.

The ETEC genome assemblies were deposited in GenBank under the accession numbers listed in Table S1.
