# Supplementary figures and images for: Genome and Functional Characterization of Colonization Factor Antigen I- and CS6-Encoding Heat-Stable Enterotoxin-Only Enterotoxigenic Escherichia coli Reveals Lineage and Geographic Variation
Source: mSystems. 2019 Jan 15;4(1):e00329-18. doi: 10.1128/mSystems.00329-18 (PMC6446980; doi:10.1128/mSystems.00329-18)

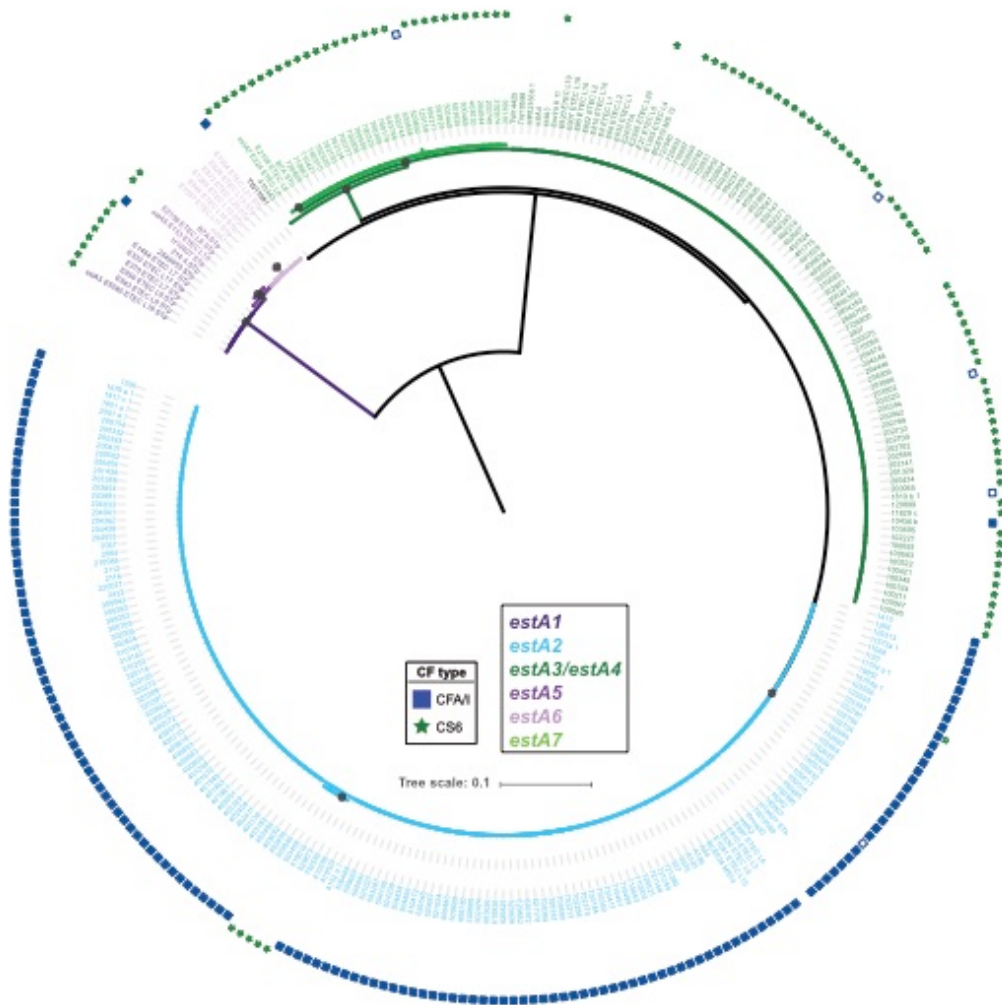

Supplement: FIG S1 [file mSystems.00329-18-sf001.pdf]

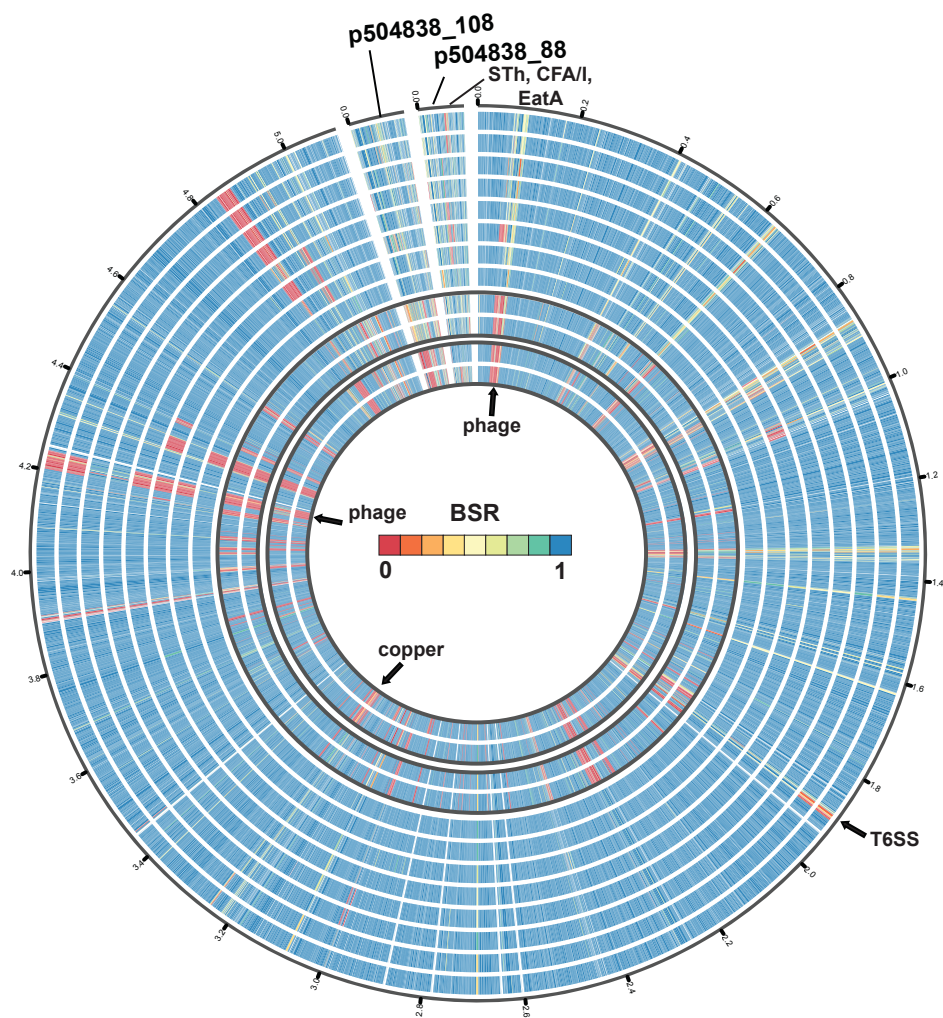

**Panel A.**

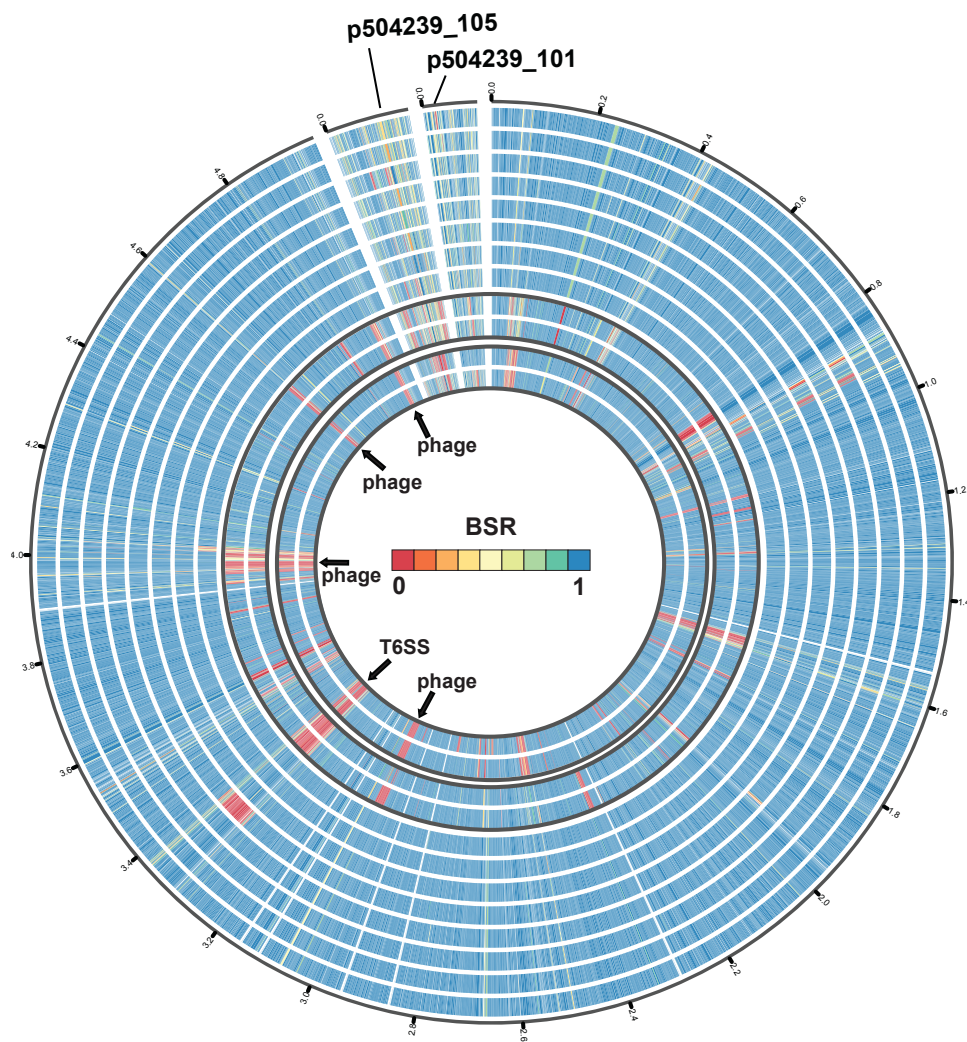

Panel B.

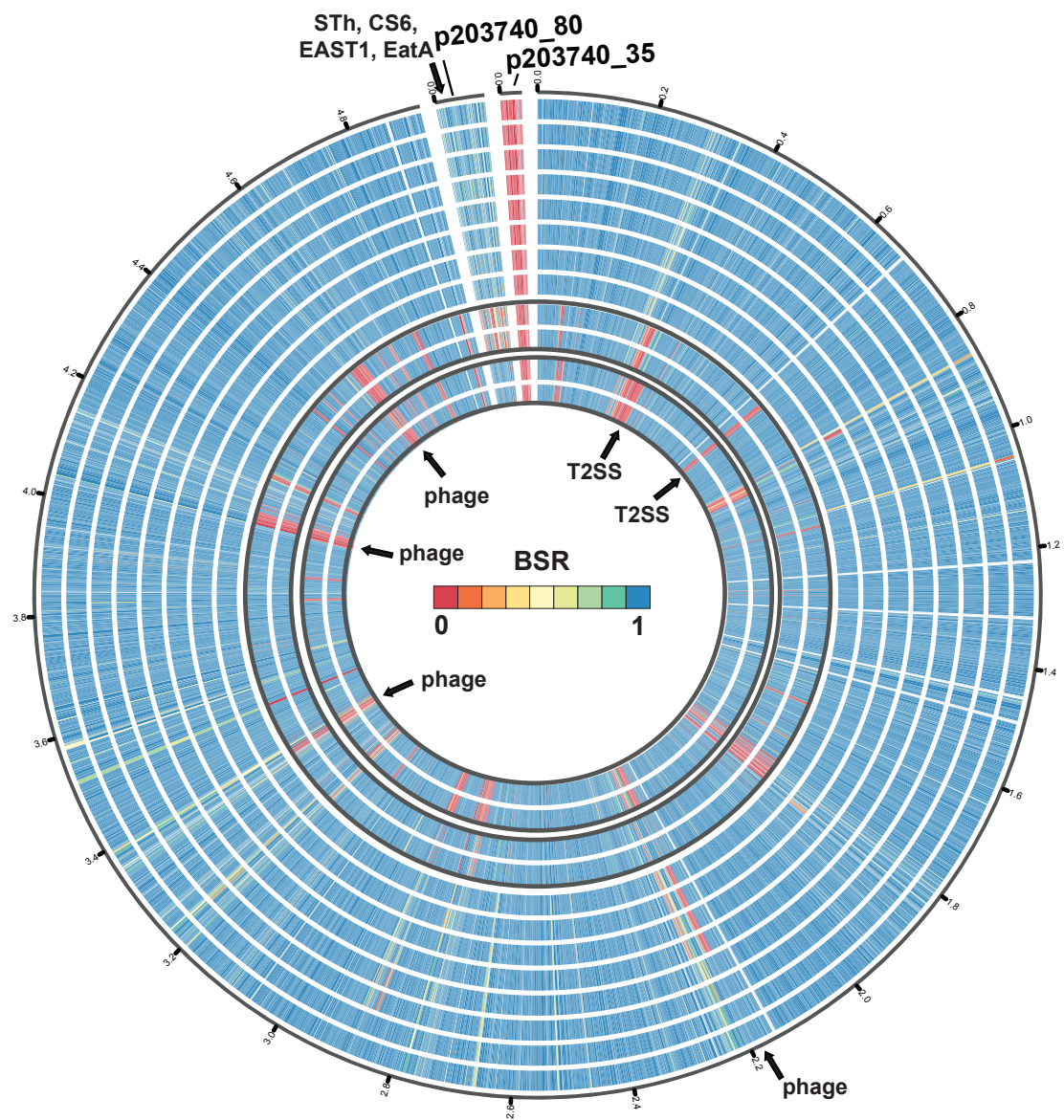

Panel C.

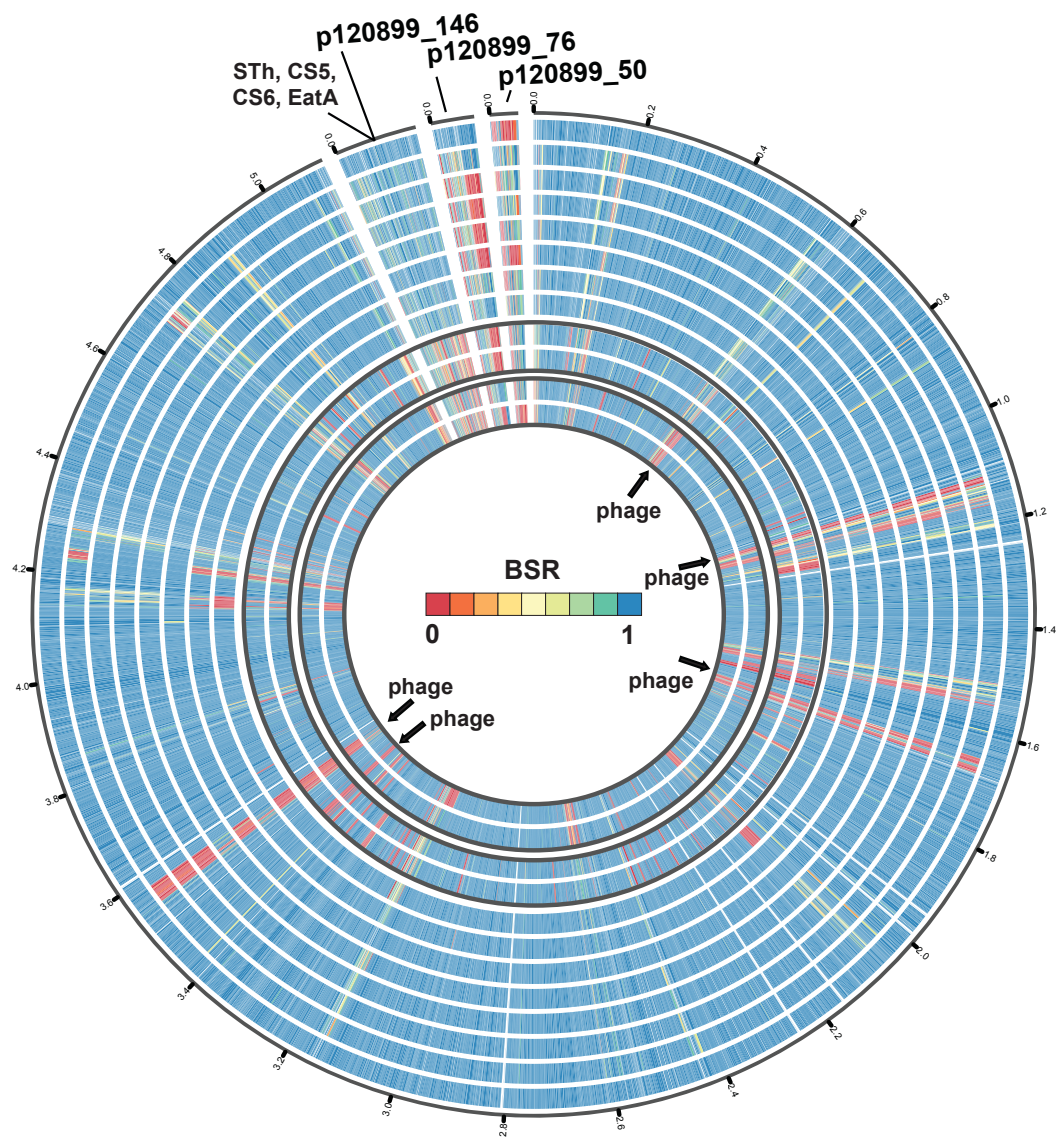

Panel D.

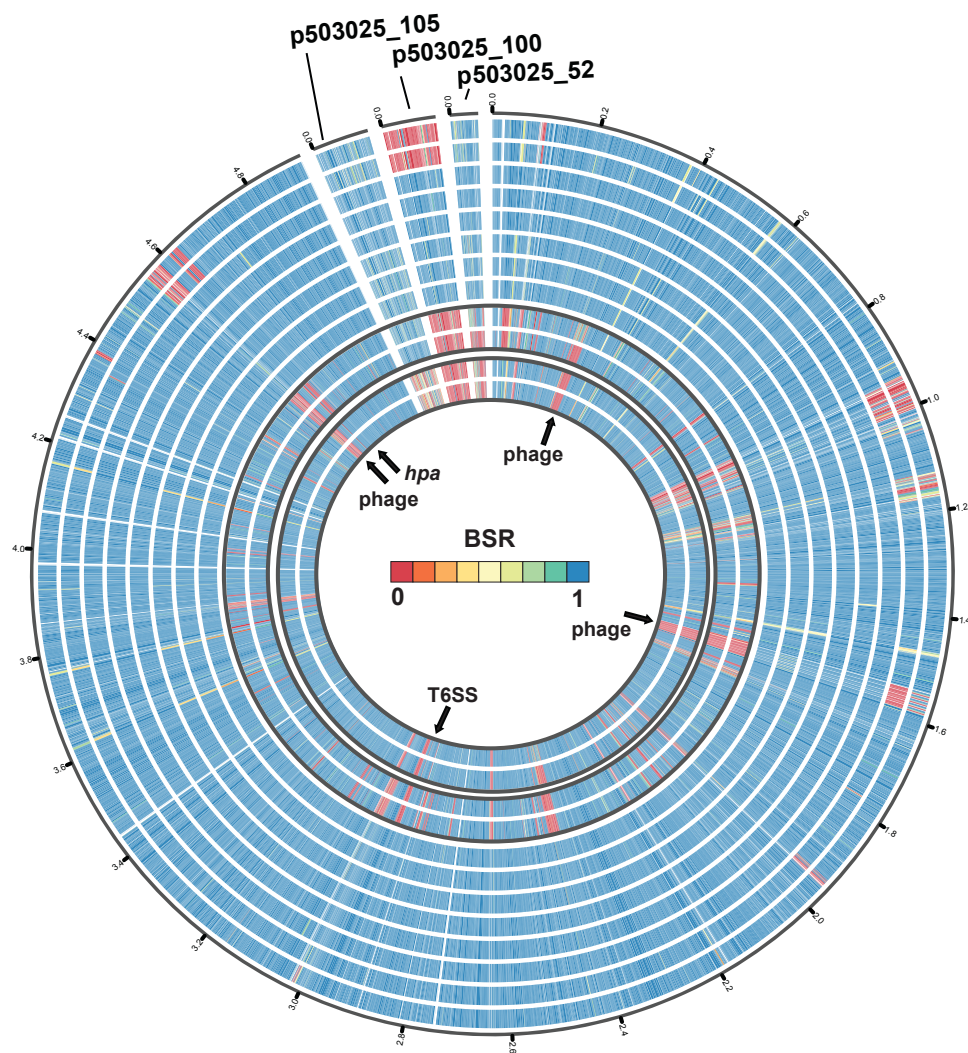

Panel E.

Supplement: FIG S2 [file mSystems.00329-18-sf002.pdf]

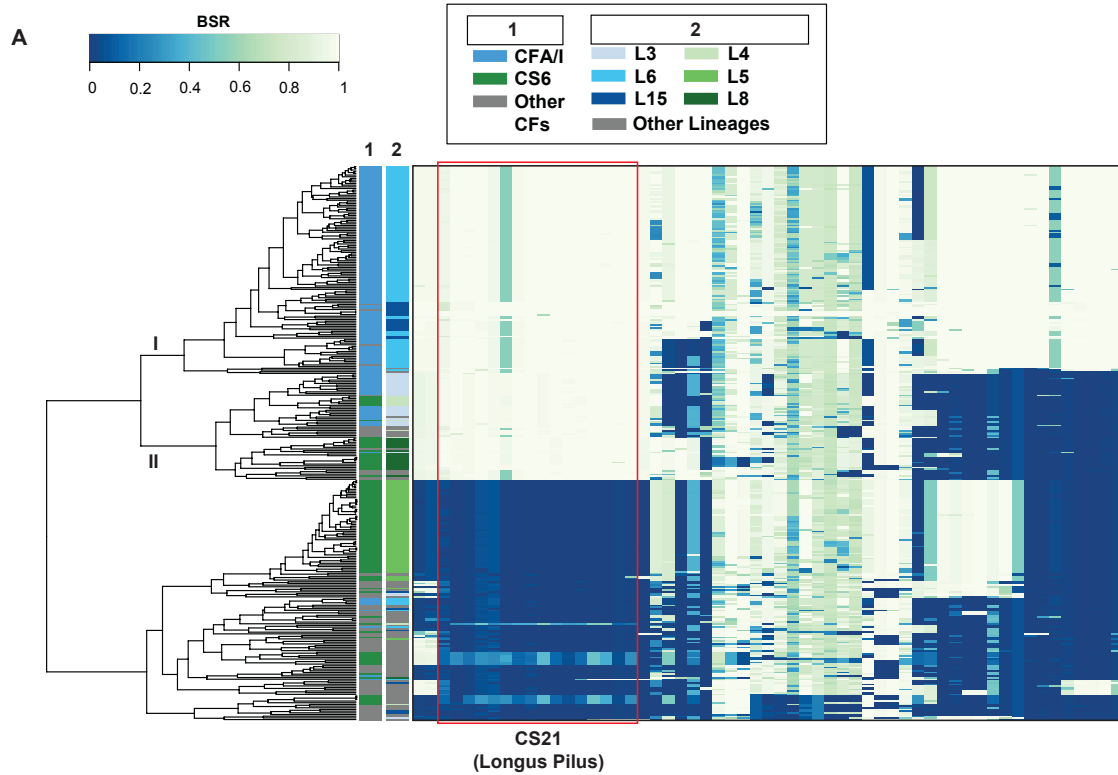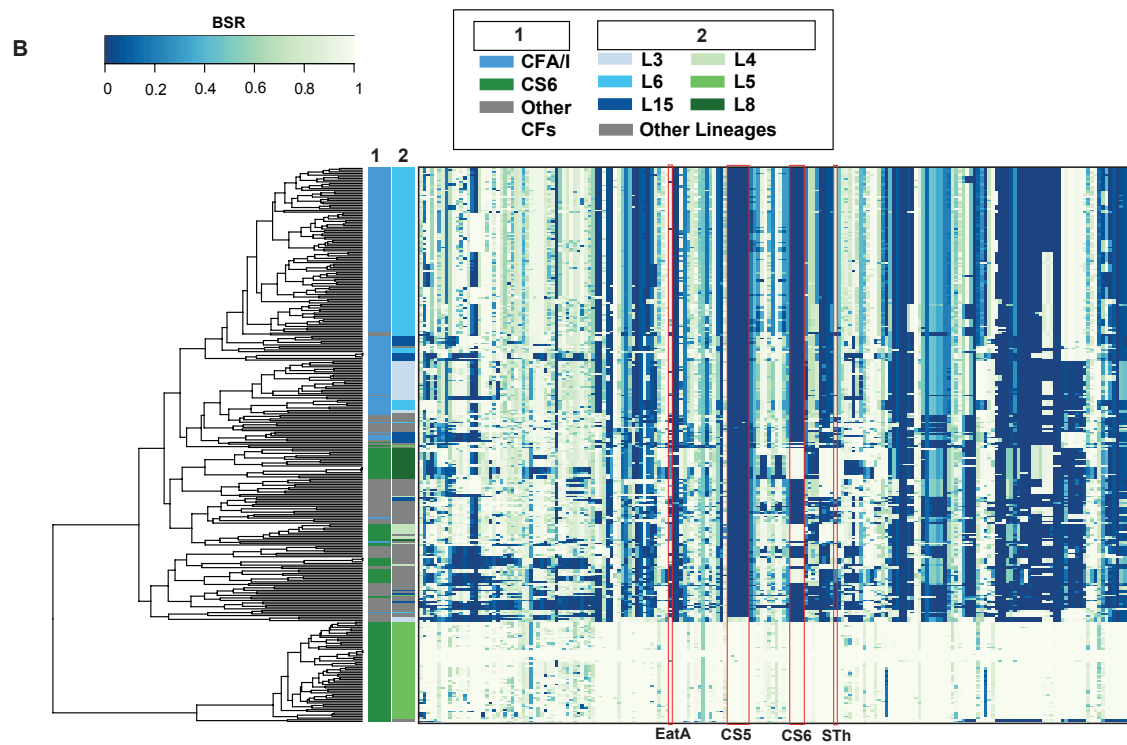

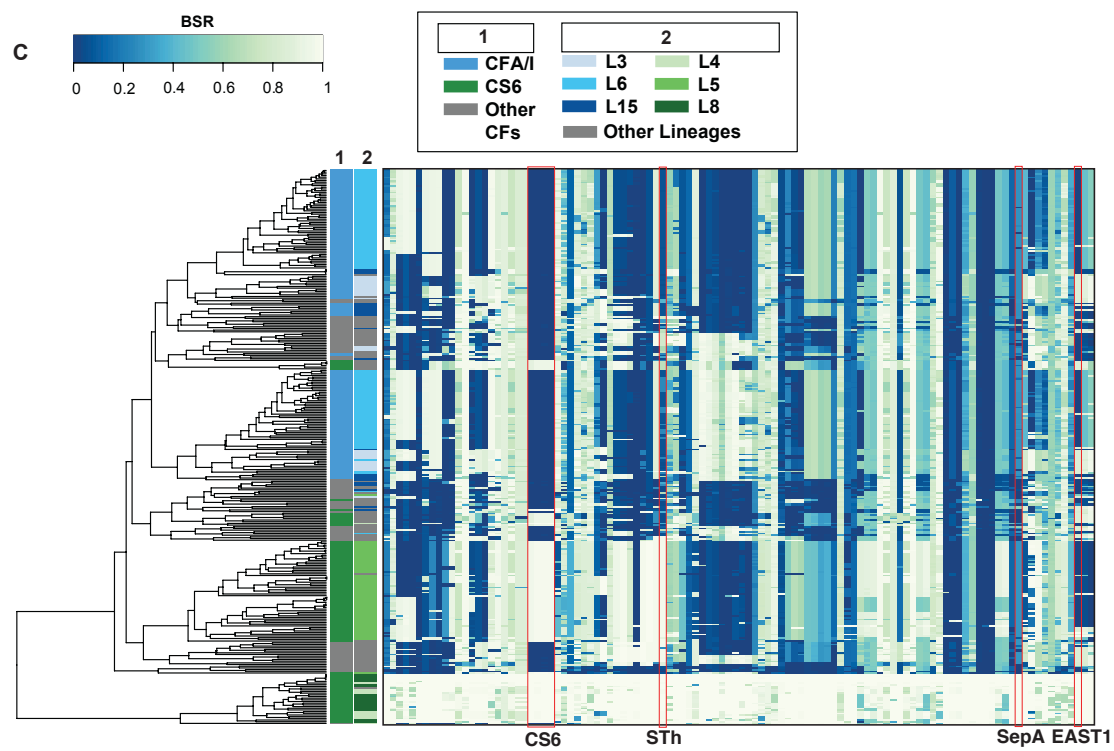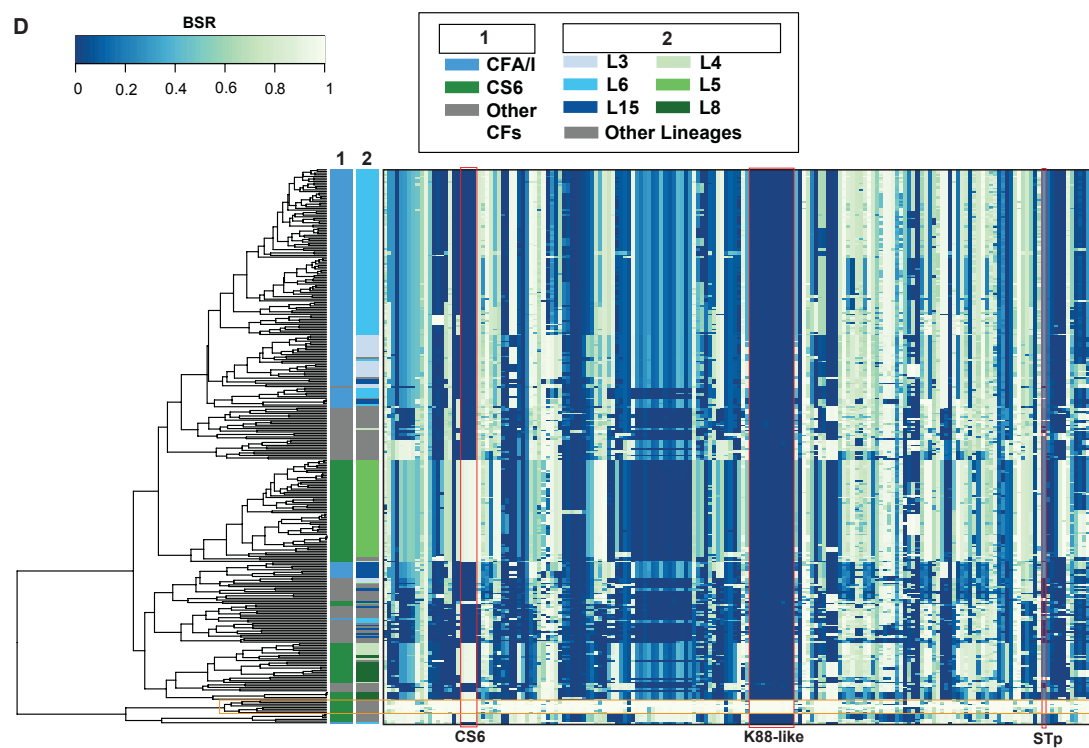

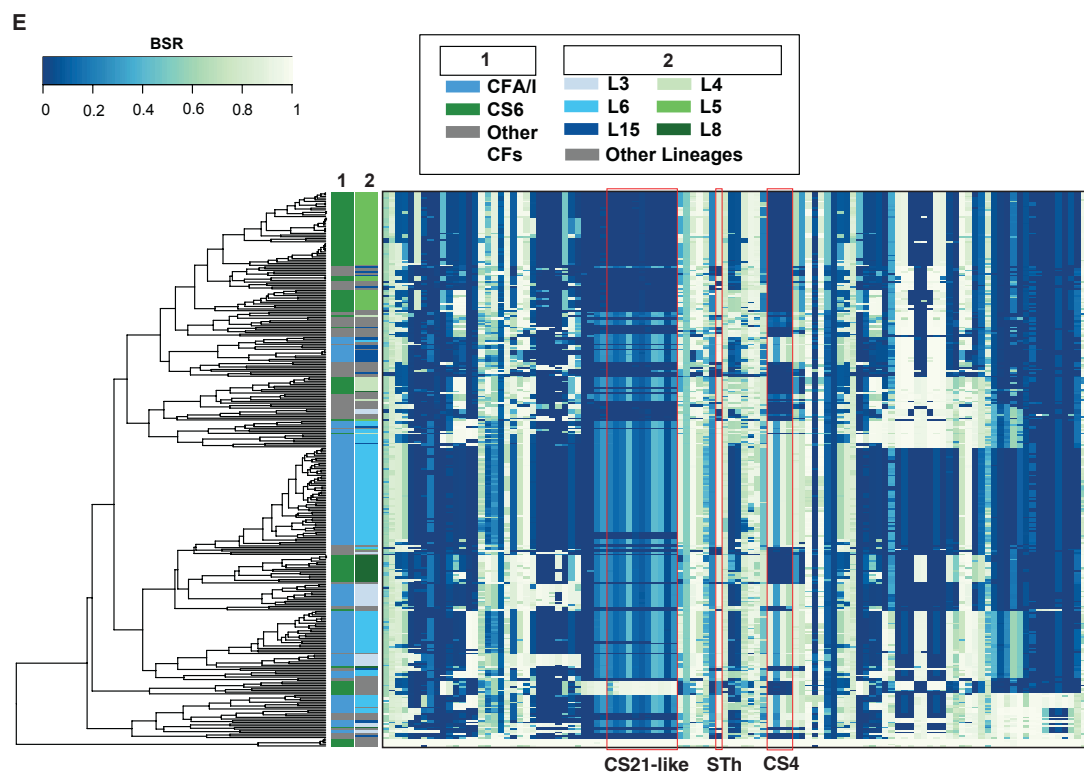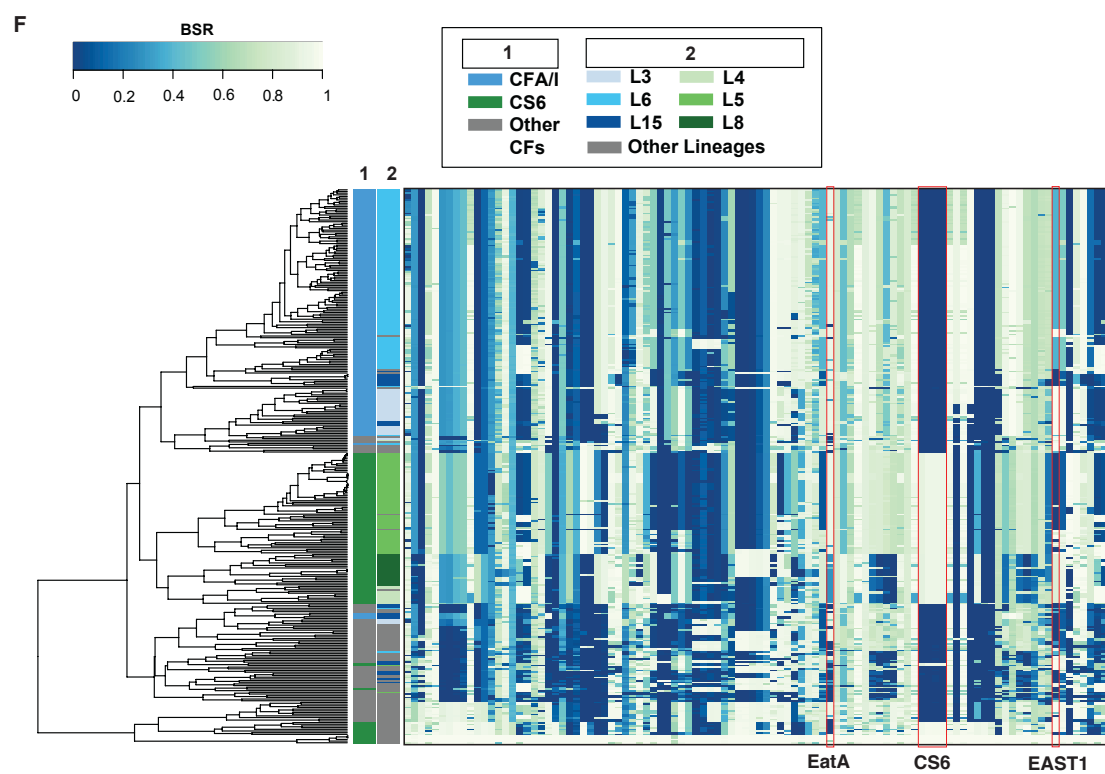

Supplement: FIG S3 [file mSystems.00329-18-sf003.pdf]
