# Supplementary material for: Genome and Functional Characterization of Colonization Factor Antigen I- and CS6-Encoding Heat-Stable Enterotoxin-Only Enterotoxigenic Escherichia coli Reveals Lineage and Geographic Variation
Source: mSystems. 2019 Jan 15;4(1):e00329-18. doi: 10.1128/mSystems.00329-18 (PMC6446980; doi:10.1128/mSystems.00329-18)
Supplement: TEXT S1 [file mSystems.00329-18-s0001.docx]

**Supplementary Information for**

Genome and functional characterization of CFA/I and CS6-encoding ST-only enterotoxigenic *Escherichia coli* reveals lineage and geographic variation

Tracy H. Hazen^1,2^, Sushma Nagaraj^1,2^ Sunil Sen^3^, Jasnehta Permala-Booth^3^, Felipe Del Canto^3^, Roberto Vidal ^3,4^, Eileen M. Barry^5,2^, Jacob P. Bitoun^6^, Wilbur H. Chen^5^, Sharon M. Tennant^5^, David A. Rasko^1,2^*

^1^Institute for Genome Sciences, ^2^Department of Microbiology and Immunology, University of Maryland School of Medicine, Baltimore, MD, USA 21201, ^3^Programa de Microbiología y Micología, Instituto de Ciencias Biomédicas, Facultad de Medicina, Universidad de Chile, ^4^Instituto Milenio de Inmunología e Inmunoterapia, Facultad de Medicina, Universidad de Chile, Santiago, Chile, ^5^Center for Vaccine Development, Institute for Global Health and Department of Medicine, University of Maryland School of Medicine, Baltimore, MD, USA 21201, ^6^Department of Microbiology & Immunology, Tulane University School of Medicine, New Orleans, LA 70112

This supplementary file 10 supplementary items including:

1 Supplementary Note, References, and Figure Legends

3 Supplementary Figures: (Figs. S1 to S3)

5 Supplementary Tables: (Tables S1 to S5)

1 Supplementary Data Sets: (S1: Clusters fasta)

**Supplementary Note**

**Additional virulence regions that exhibited a CF and lineage-specific distribution**

A T2SS has been identified in both disease-causing and non-disease-causing *E. coli* as well as other bacteria (1), and is thought to be essential for the secretion of the heat-labile enterotoxin by ETEC (2). Two distinct T2SS gene clusters, designated T2SSα and T2SSβ, were previously identified in the genomes of several ETEC including the archetype strain H10407 (3). Genes of T2SSα were identified in 81% (131/162) of the CFA/I ETEC genomes compared to only 23% (25/107) of the CS6 ETEC genomes (p-value <0.001) (Table 2). In contrast, the genes of T2SSβ were identified in 98% (159/162) of the CFA/I ETEC genomes and 81% (87/107) of the CS6 ETEC genomes (p-value <0.001) (Table 2). The genes encoding T2SS2α were identified with significant similarity in the CFA/I ETEC genomes of lineages L6 and L15 phylogroup A, but were missing from the CFA/I ETEC genomes of lineage L3 of phylogroup B1 (Fig. 3). Also, the T2SS2α genes were detected in the CS6 ETEC genomes of lineage L4 of phylogroup A, but were missing from the CS6 ETEC genomes of lineages L5 and L8 of phylogroup B1 (Fig. 3). Interestingly, while most of the CFA/I ETEC genomes had an intact T2SSβ, genomes of lineage L15 were missing or exhibited divergent sequence similarity (BSR <0.8) for one to 12 of the 14 total T2SSβ genes (Fig. 3). The T2SSβ genes were missing or divergent from five genomes of undesignated lineages in phylogroup A and one from phylogroup B1, and were completely missing from 88% (14/16) of the CS6 ETEC genomes of lineage L8 (Fig. 3). Further investigation is necessary to determine whether one or both of the T2SS may contribute to the virulence mechanisms of these ST-only ETEC.

**Comparison of representative complete ST-only ETEC genomes reveals lineage and location associated genomic variation**

Comparison of the lineage L3 CFA/I ETEC isolate 504838 with CFA/I ETEC from lineages L6 and L15 demonstrated there was variability in a region containing T6SS genes, with some of the genes exhibiting divergent sequence similarity or entirely missing from the representative genomes of the CFA/I ETEC lineages L6 and L15 (Fig. S2A). Most of the observed differences for the genome of ETEC isolate 504239 were in regions that were conserved among the L6 genomes, but were absent from the L3 and L15 genomes, including several regions containing putative phage genes (Fig. S2B, Table S5).

Comparison of the CS6-encoding ETEC isolate 203740 from lineage L4 to the other CS6 genomes demonstrated there were a number of phage regions that were conserved among the lineage L4 genomes that were absent from the genomes of lineages L5 and L8 (Fig. S2C, Table S5). The genome of CS6 ETEC isolate 120899 representing lineage L5 also exhibited geographic specificity for some of the chromosomal regions encoding phage, with genes from several regions (EC120899_1090 to EC120899_1105, and EC120899_3437 to EC120899_3482) that were also identified in another genome from Gambia (103605), but were absent from the majority of the other representative L5 genomes from other geographic locations, and were also absent from other CS6 lineage genomes (L4 and L8) (Fig. S2D, Table S5). Detection of protein-coding genes of the CS6 ETEC strain 503025 from lineage L8 demonstrated lineage-specific conservation for the genomic content of this strain, with the exception of a region with T6SS genes was identified in all of the L8 genomes but was absent from the representative genomes of lineages L4 and L5 (Fig. S2E, Table S5). Also, an *hpa* gene cluster was identified in other L8 genomes, but was missing from the L4 genomes, and also the two genomes (201399 and 403030) from L8 that were lacking genes of several phage regions (Fig. S2E, Table S5). The *hpa* gene cluster is involved in catabolism of 4-hydroxyphenylacetic acid (4), and the variability of this region among genomes from within the same lineage suggests that the ST-only ETEC strains in certain geographic locations have lost or acquired genes that may provide them with differing metabolic phenotypes.

**Three unique ST and CS6-encoding plasmids contributed to the emergence of the CS6 ST-only ETEC lineages**

The first type of STh and CS6-encoding plasmid was present in the ETEC lineage L5 genomes. The six complete CS6 ETEC genomes of ETEC lineage L5 (103605, 120899, 204446, 204576, 504237, and 602354) all had an IncFII plasmid that ranged in sequence length from 142 to 148 kb, and contained the genes encoding STh, CS5, CS6, and EatA (Table S3). *In silico* detection of the STh, CS5, CS6, and EatA-containing lineage L5 plasmid p120899_146 demonstrated that this plasmid is conserved among the CS6 ETEC of lineage L5, but was absent from CS6 ETEC of lineages L4 and L8 (Fig. S3B). Interestingly, this plasmid (p120899_146) was detected in two CS6 ETEC (2407_a and 11829_c) of lineage L17, demonstrating that this plasmid has been acquired by *E. coli* outside of ETEC lineage L5 (Fig. 1, Supplementary Fig. S3B, Table S1). The second type of CS6-encoding plasmid was identified in the genomes of lineages L4 and L8, which ranged in size from 77 to 105 kb and encoded STh, CS6, EAST1, and EatA (Table S3). *In silico* detection of the protein-coding genes of the lineage L8 plasmid p500465_77 demonstrated that this plasmid is conserved among the CS6 ETEC genomes of lineages L4 and L8 (Fig. S3C). This plasmid was also identified in the genome of ETEC isolate 202974 of lineage L11 in phylogroup A (Fig. 1, Fig. S3C). The third CS6 and ST containing plasmid was identified in the genome assembly of ETEC strain 214-4, and contained the genes encoding STp and CS6 (Table S3). *In silico* detection of p214_4_132 demonstrated this plasmid was present in seven previously sequenced ETEC genomes in addition to ETEC strain 214-4 (Fig. S3D, horizontal orange box). Interestingly, p214_4_132 contained a novel fimbrial region that was absent from all of the other ETEC genomes analyzed (Supplementary Fig. S3D). However, these genes had conserved organization and 99 to 100% nucleotide identity to the genes of a K88-like fimbrial gene cluster previously identified on plasmid pEntYN10 (GenBank accession number [AP014654.2](https://www.ncbi.nlm.nih.gov/sites/entrez?cmd=Search&db=nucleotide&term=AP014654.2&dopt=GenBank)) from ETEC O169:H41 strain YN10 (5). The K88-like genes of pEntYN10 and p214_4_132 had similar organization, but divergent sequence similarity (27 to 64% amino acid identity) to the genes of K88 from plasmid pUMNK88_K88 from the porcine ETEC strain UMNK88 (6).

**A conserved antimicrobial resistance plasmid was identified in the complete CS6-producing ST-only ETEC**

Although the 26 ETEC isolates selected for genome completion were susceptible to the eight antibiotics analyzed (Table S4), the genome assemblies of five ETEC isolates contained a plasmid with known antibiotic resistance genes (Table S3). There was an identical 36,521 bp IncX4 plasmid in the CS6 ETEC isolates 503458, 510016, and 520873 that contained *sat-1* and *drfA25,* which confer resistance to streptothricin (7) and trimethoprim (8), respectively (Tables S3 and S4). All three of the isolates were lacking a gene for resistance to sulfamethoxazole, which may have contributed to their susceptiblity to trimethoprim-sulfamethoxazole (Table S4). A 79,888 bp IncFII(pCoo) plasmid was identified in the genome assembly of CS6 ETEC isolate 500465 that contained *qnrS1* and *bla*_TEM-1_ (Table S3). Although *qnrS1* typically confers resistance to fluoroquinolones such as ciprofloxacin and levofloxacin (9), ETEC isolate 500465 was susceptible to both antibiotics, suggesting *qnrS1* may not be functional in this ETEC isolate (Table S4). The genome assembly of the CS6 ETEC isolate 720632 had a 72,294 bp IncFIB(K) plasmid with *sul2*, *qnrS1, bla*_TEM-1_, *aph(3'')-Ib,* and *aph(6)-Id* (Table S3). Although ETEC isolate 720632 contained *qnrS1,* similar to ETEC isolate 500465, it was also susceptible to the fluroquinolone antibiotics ciprofloxacin and levofloxacin (Table S4). Also, ETEC isolate 720632 contained *sul2,* which can confer resistance to sulfonamides such as sulfamethoxazole (10, 11), but was lacking a gene for trimethoprim resistance, and was susceptible to trimethoprim-sulfamethoxazole (Table S4). Thus, by completing the genomes of select ST-only ETEC isolates, we identified antibiotic resistance plasmids in several of the genomes that contain genes that may confer reduced susceptibility to some clinically-relevant antibiotics, which can be used to inform selection of these ST-only ETEC isolates for future functional studies or clinical trials.

**ST-only ETEC carry a wide array of antimicrobial resistance genes**

The ETEC strains analyzed by whole-genome sequencing were also tested for their susceptibility to eight frequently-prescribed antibiotics, using the Kirby-Bauer disk diffusion test (Table S4). These antibiotics were selected to be representative of the major classes of antimicrobials that might be frequently used in the local respective populations and for which there may be pre-existing resistance. The percentage of the 269 ST-only ETEC isolates that exhibited some level of antibiotic resistance ranged from 3% (9/269) with resistance to azithromycin to 77% (203/269) with resistance to trimethoprim-sulfamethoxazole (Table S4). *In silico* detection of known antibiotic resistance genes in the genome assemblies of all 269 ST-only ETEC isolates demonstrated that 90% (247/269) of the strains contained one or more known antibiotic resistance genes, including genes known to confer resistance to antibiotics other than the eight antibiotics that were included in the susceptibility testing (Table S4). Among the detected antibiotic resistance genes were *bla*_CTX-M-15_, *sul1, dfrA7, aph(3'')-Ib,* and *qnrS1*, which encode proteins that confer resistance to cephalosporins, sulfonamides, trimethoprim, aminoglycosides, and fluoroquinolones, respectively (11-13)(Table S4). Twenty-six of the ST-only ETEC genomes contained the gene encoding CTX-M-15, which is an extended-spectrum β-lactamase (ESBL) that has been demonstrated to confer resistance to cephalosporins, such as ceftriaxone (12). The antimicrobial susceptibility testing confirmed that all of the *bla*_CTX-M-15_-containing strains exhibited intermediate or resistant growth to ceftriaxone (Table S4). A dihydrofolate reductase gene (*dfrA)*, which typically confers resistance to trimethoprim (14), was identified in 73% (196/269) of the ST-only ETEC genomes (Table S4). Among the other resistance genes detected were *tet(A), tet(D),* and *tet(G),* which encode efflux pumps that confer resistance to tetracycline (15). One or more of the *tet* genes were identified in 64% (173/269) of the ST-only ETEC genome assemblies, while 65% (173/269) of the ST-only ETEC strains exhibited resistance to tetracycline (Table S4).

**REFERENCES**

1. Patrick M, Gray MD, Sandkvist M, Johnson TL. 2010. Type II Secretion in *Escherichia coli*. EcoSal Plus 4.

2. Tauschek M, Gorrell RJ, Strugnell RA, Robins-Browne RM. 2002. Identification of a protein secretory pathway for the secretion of heat-labile enterotoxin by an enterotoxigenic strain of *Escherichia coli*. Proc Natl Acad Sci U S A 99:7066-71.

3. Strozen TG, Li G, Howard SP. 2012. YghG (GspSbeta) is a novel pilot protein required for localization of the GspSbeta type II secretion system secretin of enterotoxigenic *Escherichia coli*. Infect Immun 80:2608-22.

4. Prieto MA, Diaz E, Garcia JL. 1996. Molecular characterization of the 4-hydroxyphenylacetate catabolic pathway of *Escherichia coli* W: engineering a mobile aromatic degradative cluster. J Bacteriol 178:111-20.

5. Ban E, Yoshida Y, Wakushima M, Wajima T, Hamabata T, Ichikawa N, Abe H, Horiguchi Y, Hara-Kudo Y, Kage-Nakadai E, Yamamoto T, Wada T, Nishikawa Y. 2015. Characterization of unstable pEntYN10 from enterotoxigenic Escherichia coli (ETEC) O169:H41. Virulence 6:735-44.

6. Shepard SM, Danzeisen JL, Isaacson RE, Seemann T, Achtman M, Johnson TJ. 2012. Genome sequences and phylogenetic analysis of K88- and F18-positive porcine enterotoxigenic *Escherichia coli*. J Bacteriol 194:395-405.

7. Heim U, Tietze E, Weschke W, Tschape H, Wobus U. 1989. Nucleotide sequence of a plasmid born streptothricin-acetyl-transferase gene (sat-1). Nucleic Acids Res 17:7103.

8. Agerso Y, Peirano G, Aarestrup FM. 2006. *dfrA25*, a novel trimethoprim resistance gene from *Salmonella* Agona isolated from a human urine sample in Brazil. J Antimicrob Chemother 58:1044-7.

9. Hata M, Suzuki M, Matsumoto M, Takahashi M, Sato K, Ibe S, Sakae K. 2005. Cloning of a novel gene for quinolone resistance from a transferable plasmid in *Shigella flexneri* 2b. Antimicrob Agents Chemother 49:801-3.

10. Radstrom P, Swedberg G. 1988. RSF1010 and a conjugative plasmid contain sulII, one of two known genes for plasmid-borne sulfonamide resistance dihydropteroate synthase. Antimicrob Agents Chemother 32:1684-92.

11. van Hoek AH, Mevius D, Guerra B, Mullany P, Roberts AP, Aarts HJ. 2011. Acquired antibiotic resistance genes: an overview. Front Microbiol 2:203.

12. Canton R, Gonzalez-Alba JM, Galan JC. 2012. CTX-M Enzymes: Origin and Diffusion. Front Microbiol 3:110.

13. Roberts MC, Schwarz S, Aarts HJ. 2012. Erratum: Acquired antibiotic resistance genes: an overview. Front Microbiol 3:384.

14. Stone D, Smith SL. 1979. The amino acid sequence of the trimethoprim-resistant dihydrofolate reductase specified in Escherichia coli by R-plasmid R67. J Biol Chem 254:10857-61.

15. Roberts MC. 2005. Update on acquired tetracycline resistance genes. FEMS Microbiol Lett 245:195-203.

16. Kumar S, Stecher G, Tamura K. 2016. MEGA7: Molecular Evolutionary Genetics Analysis Version 7.0 for Bigger Datasets. Mol Biol Evol 33:1870-4.

17. Sahl JW, Caporaso JG, Rasko DA, Keim P. 2014. The large-scale blast score ratio (LS-BSR) pipeline: a method to rapidly compare genetic content between bacterial genomes. PeerJ 2:e332.
